# Effects of plate interface frictional heterogeneities on earthquake cycle dynamics in subduction zones

**DOI:** 10.1038/s41598-026-43399-7

**Published:** 2026-04-01

**Authors:** Sayak Ray, Abhijit Ghosh, Bhaskar Kundu, Batakrushna Senapati

**Affiliations:** 1 Department of Earth and Atmospheric Sciences, NIT Rourkela, Rourkela, 769008 India; 2 Department of Earth and Planetary Sciences, University of California, Riverside, 900 University Ave, Riverside, CA 92521 USA; 3 Department of Earth Science, National Central University, No. 300, Jhongda Rd., Chungli, Taoyuan, 320 Taiwan

**Keywords:** Natural hazards, Solid Earth sciences

## Abstract

Subducting topographic features—including seamounts and ridges—introduce frictional complexities that can affect megathrust seismicity. Whether these heterogeneities tend to facilitate large earthquakes by acting as asperities or impede rupture propagation as barriers remains debated. To address this, we combine laboratory stick-slip experiments with quasi-dynamic numerical simulations to investigate how frictional barriers affect earthquake cycles. The experiments show that increasing the area of a single circular barrier within an asperity leads to a transition from periodic stick-slip (seismic events) to aseismic slip when the barrier area exceeds a threshold between 8 and 11% of the apparent contact area of the sample. For multiple barriers, the orientation relative to the loading direction strongly influences slip behaviour, with linear arrangements perpendicular to loading promoting a mix of seismic and aseismic slip, while parallel arrangements predominantly exhibit aseismic behaviour. Numerical simulations of a synthetic segmented fault and the Alaska-Aleutian subduction zone show that a velocity-strengthening patch acts as a permanent seismic barrier when its along-strike length constitutes 0.38–0.43 of the fault segment. This range aligns with—and extends slightly beyond—the threshold from our laboratory experiments, where the dimensionless ratio L_b_/L falls between 0.32 and 0.38. In contrast, the subsurface ridges along the Himalayan deformation front are too small to act as permanent barriers, allowing dynamic ruptures to propagate across them under favourable conditions. The consistency of this dimensionless parameter across methods provides a robust metric for evaluating barrier potential in subduction zones. Our results align with previous dynamic rupture models, which show that stress transfer and fault rheology facilitate cascading ruptures. While paleoseismic data provide snapshots of past events, numerical modelling reveals physically plausible scenarios beyond current empirical constraints. Collectively, these findings suggest that the role of frictional barriers in modulating earthquake behaviour depends not only on their size but also on their geometry and spatial distribution relative to the plate velocity. These findings have significant implications for understanding objective seismic hazards in subduction zones characterised by frictionally heterogeneous plate interfaces.

**Supplementary Information:**

The online version contains supplementary material available at 10.1038/s41598-026-43399-7.

## Introduction

Subduction zones, which often host large interplate earthquakes (M ≥ 7.5), account for ∼90% of the total global seismic moment release and have been a focal point of scientific research in the past few decades^1^. Previous studies have recognized that the intricacies of subduction zone earthquakes, their magnitude, spatiotemporal irregularity of occurrence and/or quiescence are primarily governed by the degree of seismic coupling along the plate interface^2^. There have been many efforts to explain variations of seismic coupling in terms of general tectonic factors, for example, age of the subducting plate and convergence rate^2–5^, motion of the upper plate^4,6,7^, quantity of sediment subducted^8^ and temperature conditions in the thrust zone^9–11^. Such general tectonic factors, or their combinations, have been partially successful in explaining broad features like the seismic coupling ratio, maximum earthquake magnitude, and differences in coupling width; however, they fail to account for the complexity of subduction zone seismicity on regional or local scales. For example, (1) why do young subducting oceanic plates of similar ages (∼10 Ma), in Cascadia, Mexico and southwest Japan have different recurrence intervals of seismic activity? (2) why do large earthquakes frequently occur in the northern segment of the Japan Trench but are a rarity in the southern segment^12–14^? or (3) in general, why do some areas of megathrust frequently release stress through large seismic slips, while others are mostly aseismic or creeping and remain silent for hundreds of years?

Such characteristic non-uniformity and outright unpredictability of megathrust seismicity could only be attributed to spatially heterogeneous plate interface or fault strength. To elucidate the complexity of large events, the asperity model, an outgrowth of rock friction laboratory experiment results^15,16^ was first implemented by Kanamori^17^ and subsequently developed based on detailed analysis of source rupture process^18–20^. The model successfully explained variations in seismic moment release, the concentration of slip, high-frequency radiation during earthquakes and earthquake recurrence^17,20^. However, it faced limitations in accounting for dynamic rupture complexities, such as interactions between multiple asperities and the evolution of fault strength over multiple earthquake cycles^21–23^. Additionally, several characteristics of source complexity in large events and their related seismicity patterns have been analysed and explained using the barrier model^24,25^. While it provided a compelling explanation for the termination of ruptures and the generation of aftershocks, it struggled to fully account for the dynamic processes governing rupture initiation and propagation. Often, the distinction between the two models (asperity and barrier) can be a source of confusion; nevertheless, both models share the basic tenet of non-uniform stress along a fault, with localized zones of stress concentration dictating the mode of failure^26^.

It is well established that bathymetric or topographic features of the seafloor, such as seamounts, aseismic ridges, fractures, and rises, influence earthquake nucleation, rupture propagation, and the location and frequency of great earthquakes along the subduction zones^27–31^. Thus, seafloor roughness plays a crucial role in friction of the plate interface, hence seismic behavior^32^. These features, particularly seamounts, may act as (1) asperities^33–36^ promoting large earthquakes by increasing the normal stress across the subduction interface^37,38^ or as (2) barriers by inhibiting ruptures^39–45^ or promoting smaller earthquakes and creeping by creating fracture networks^44,46–49^. Sun et al.^50^ discussed how highly porous sediments and the presence of fluids atop seamounts reduce fault strength and promote aseismic, slow slip. The Nazca Ridge in the Peru–Chile trench is probably the best example seismic barrier that affects the ruptures of great and major earthquakes^30,51^. Primarily these features contribute to plate interface roughness upon subduction and are widely accepted to influence megathrust earthquakes^52–54^. Recent studies from natural observations of seafloor data around the Ring of Fire^55,56^ and experimental analogue models of earthquakes^57^ converge towards the outcome that statistical correlation exists between areas of smooth seafloor and the occurrence of great earthquakes and that interplate roughness reduces the megathrust interseismic coupling^57^. Over the years, various subduction zone parameters have been identified to explain this, as reviewed by Lallemand and Heuret^58^.

**Fig. 1:**
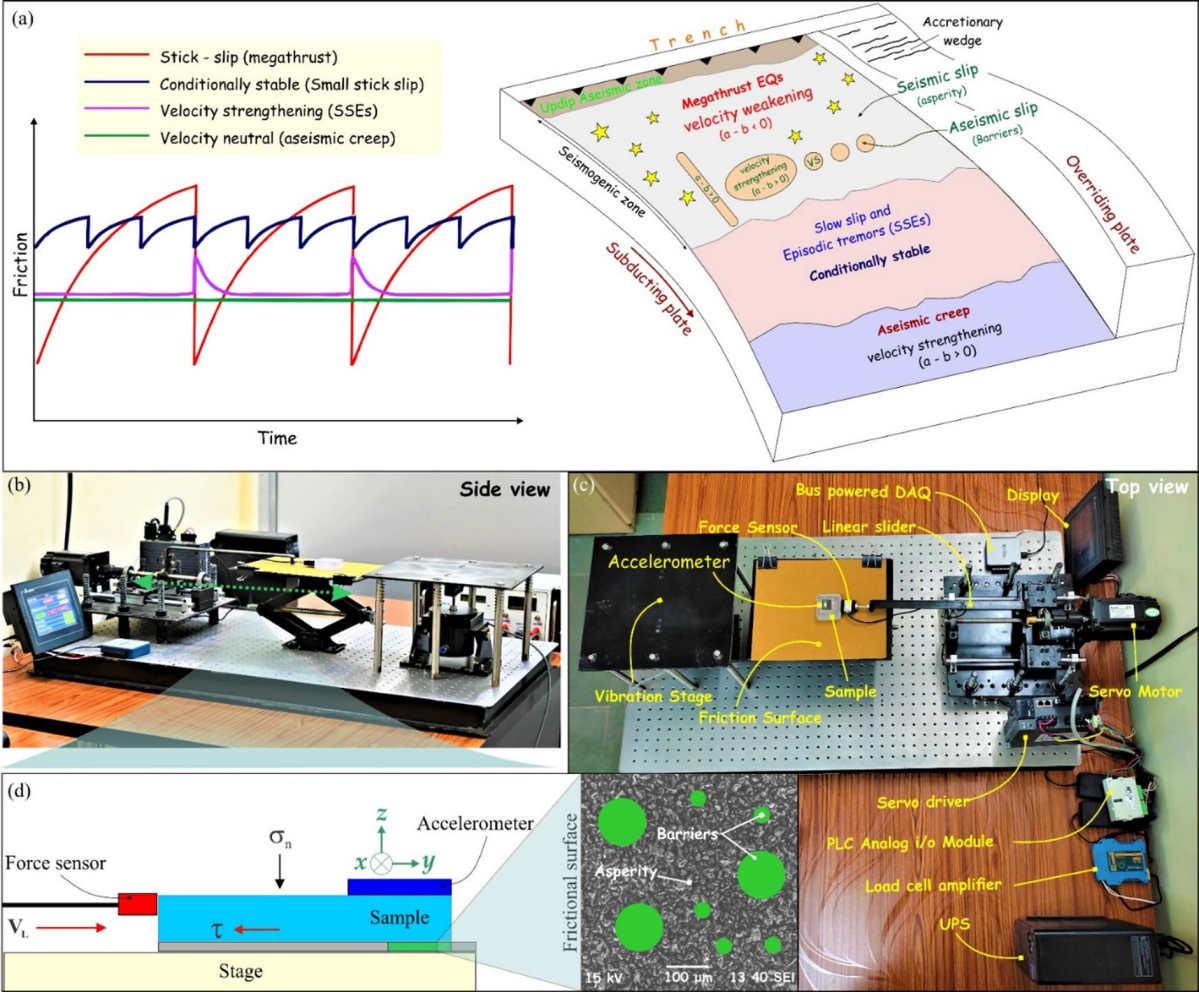
(**a**) Schematic diagram of idealised frictional response (left, modified after Kosari et al.^70^) and conceptual model of heterogeneous seismic/aseismic behaviour of a megathrust fault (right). It is divided into a shallow updip zone of aseismic slip followed by a velocity-weakening zone (asperity) downdip, where Megathrust earthquakes (indicated by the yellow stars) take place. In the seismogenic zone, the presence of various types of velocity-strengthening patches results in slow slip/episodic tremors. Further downdip is the ductile zone which is dominated by aseismic creep. Laboratory setup of the stick-slip experiment, (**b**) side view (**c**) top view with all the components marked. (**d**) Schematic illustration showing kinematics of friction and primary data acquisition system with a schematic frictional surface enlarged to the right, containing asperities and barriers. CorelDRAW (version 18; URL: https://www.coreldraw.com/en/) has been used to generate this figure.

Subduction zone thrust faults, such as those in Cascadia, Nankai, and Alaska-Aleutian, are often characterised by a seismogenic zone that extends approximately 5–10 km to 30–70 km downdip, where the fault remains locked during the interseismic period. This locked region (velocity weakening, a – b < 0) is bounded both updip and downdip by segments (velocity strengthening, a – b > 0 or conditionally stable, a – b = 0) that primarily deform aseismically through stable sliding or creep^21,59–61^ (Fig. 1a). Most megathrust earthquake ruptures occur within the shallow seismogenic zone, typically extending from 0 to 50 km depth. Within this depth range, seismic and geodetic observations reveal complex slip patterns, with the plate interface composed of interfingered patches that exhibit contrasting behavior: some remain locked during the interseismic period and eventually rupture seismically, while others creep aseismically at low rates without radiating seismic energy (Fig. 1a). This along-strike segmentation exerts a strong influence on rupture behavior^62–68^: locked patches may break independently or cascade into multi-segment failures, generating earthquakes of diverse magnitudes.

Predominantly creeping patches that are characterised by a velocity strengthening frictional behaviour can act as systematic barriers to earthquake propagation, as in the case of the Batu Islands area, Sumatra^64^, or in the Paracas Peninsula area, Southern Peru^66^. Therefore, along-strike changes in kinematic coupling, segmentation and deformation with respect to seismogenic behaviour of a subduction zone is crucial because the size of the most hazardous, large earthquakes scales with the length of the ruptured segment. Corbi et al.^69^ implemented a 3D laboratory analogue model simulating along-strike rupture behaviour of megathrust earthquakes using velocity weakening asperities and velocity strengthening barriers and compared their results to Nankai Trough historical seismicity to explain the diversity of earthquakes therein. Kosari et al.^70^ used a similar analog seismotectonic model of an elastoplastic wedge overlying a frictionally heterogeneous megathrust to investigate the along-strike segmentation effect on seismogenic megathrust.

Subduction zones that are characterised by a very rough (e.g., Mariana type) or very smooth (e.g., Kuril type) subducting seafloor. Significant along-strike changes in kinematic coupling have been recognised along worldwide subduction zones, including Alaska^71^, Sumatra^64^, Japan^72^, the Himalaya^73^, Peru and Chile^66^. The Alaska–Aleutian megathrust plate interface has experienced several great earthquakes over the last century (e.g. 1938, 1946, 1957, 1964, 1965 and 2021), although some notable gaps remain^74,75^. The Shumagin seismic gap in the Alaska Peninsula is one such prominent unruptured segment lying between the 1946 M8.6 earthquake in the southwest Unimak segment and the 1938 M8.2 earthquake in the Semidi segment^76^. Geodetic studies indicate the Semidi segment is currently locked^77^. The combined seismic, geodetic, and geologic evidence strongly suggests low plate interface coupling in the Shumagin Gap that slips aseismically^78^. It acts as a seismic barrier (along-strike) that limits earthquakes with magnitudes Mw > 8.0^79^. The Shumagin segment’s behaviour is inconsistent with the rest of the Alaska-Aleutian subduction zone and therefore requires significant attention.

Understanding the partitioning between seismic (asperities) and aseismic slip/creep (barriers) regions is an important issue in seismotectonics, since it is a key factor in determining observed patterns of seismic ruptures and the seismic potential in subduction zones, an issue that is the focus of this study. Traditional hazard prediction methods often fail to identify areas prone to the largest earthquakes^80^, as digital earthquake catalogues span only ~ 100 years, far shorter than the recurrence intervals of great earthquakes^81^. While continuous observations of repetitive seismic phenomena offer insights into their nature and origin, the limited modern instrumental record of multiple cycles of large earthquakes is usually supplemented by employing systematic laboratory analogue experiments and numerical simulation studies. Despite their unavoidable experimental oversimplifications, they are advantageous for physically self-consistent behaviour and the capability of reproducing tens of seismic cycles in a convenient experimental time^69^.

Here, it is important to clarify the terminology central to this study. In natural subduction zones, ‘plate interface roughness’ typically refers to large-wavelength (kilometer-scale) bathymetric features such as seamounts and ridges. The subduction of such features is thought to generate frictional heterogeneities—patches with velocity-weakening (seismic) or velocity-strengthening (aseismic) behavior. Our laboratory experiments do not replicate the large-scale geometry of these topographic features. Instead, we use surfaces with contrasting small-scale (micrometer) roughness to create controlled frictional contrasts that simulate the seismic/aseismic dichotomy. Therefore, while natural seafloor roughness may be one origin of frictional heterogeneity, our experiments focus on the fundamental effects of the resulting frictional contrast on earthquake cycle dynamics. Here we implement single-degree-of-freedom spring-block stick-slip experiments on a frictionally heterogeneous surface, where barriers are embedded within asperities. Due to semantic shift, ‘barriers’ and ‘asperities’ vary in usage across contexts. The next section reviews their definitions in previous studies and clarifies their application in the present study. We perform several laboratory experiments to investigate the effect of frictional contrast on stick-slip cycles (analogous to earthquake cycles), particularly focusing on how the area, geometry and distribution of seismic barrier (VS) contribute to the occurrence of seismic and aseismic behaviour. Further, we correlate our laboratory findings with QuasiDYNamic Simulation of earthquakes cycles assuming (i) a hypothetical (we call it synthetic) fault with along strike segmentation; scaled rupture segments of (ii) Alaska Aleutian subduction zone containing the Shumagin gap as a seismic barrier bounded by velocity weakening segments on either side and (iii) Himalaya deformation front containing the three velocity strengthening ridges. The details of the laboratory experiments and the methodology for numerical simulations carried out in this study are described under Materials and Methods.

## Contextual usage of ‘barriers’ and ‘asperities’

We note that the terms ‘barrier’ and ‘asperity’ are widely used in a variety of contexts. To the kinematic modelling community, asperities are regions of high slip. Some authors interpret asperity as a region that remained unbroken during a previous earthquake, therefore, has a higher prestress than the surrounding fault, while defining a barrier as a region that has a higher frictional strength (yield stress) than the surrounding fault^82–84^. Lay and Kanamori^85^ proposed that asperities are strong patches with high relative stress where two fault blocks have a high frictional strength, which leads to fault locking at these locations. In contrast, based on strength relative to applied stress, others view barriers as a strong section of the fault or unbroken patches on a fault surface after an earthquake that make rupture propagation irregular, slow it down and cause repeated stick-slip. They are not only stoppers but also initiators of rupture as well as stress concentrators^24,25,86^. Aki^25^ proposed that barriers can be linked either to geometrical irregularities of the fault, like bending or segmentation, or to the heterogeneity of rock physical properties along the fault (geometric and relaxation barriers^86,87^. In the framework of rate-state friction, asperities are characterised by velocity-weakening frictional behaviour (i.e., the friction rate parameter a–b < 0), indicating that seismic rupture may nucleate and easily propagate, while barriers are characterised by velocity-strengthening frictional behaviour (i.e., a–b > 0) that inhibits the seismic rupture propagation^21^. Recent studies view potential local barriers in the form of a fault region with velocity-strengthening friction or creeping barriers^65,88,89^. In our study, asperity represents a frictionally strong region locked interseismically and velocity-weakening (hereafter, VW) friction that releases strain in the form of large stick slips (or seismic slips)^62^. In contrast, barriers are characterised by frictionally weak regions with minimal interseismic coupling and velocity-strengthening (VS) friction that accommodate interseismic strain release through slow/aseismic slip or creeping (Fig. 1a). In natural subduction zones, such frictional heterogeneities (VS barriers and VW asperities) can originate from variations in lithology, fluid pressure, temperature, or the presence of subducted seafloor features (e.g., seamounts, ridges). Our laboratory experiments simulate these frictional contrasts using surfaces with different small-scale roughness, which produce analogous stick-slip (seismic) and stable sliding (aseismic) behaviors. Thus, while we refer to ‘roughness’ in the context of our measured surface parameters (Ra, Rq, see supplementary), the essential physical mechanism under investigation is the frictional contrast between velocity-weakening and velocity-strengthening regions.

## Results

### Laboratory-based analogue experiments with frictional surfaces

**Fig. 2:**
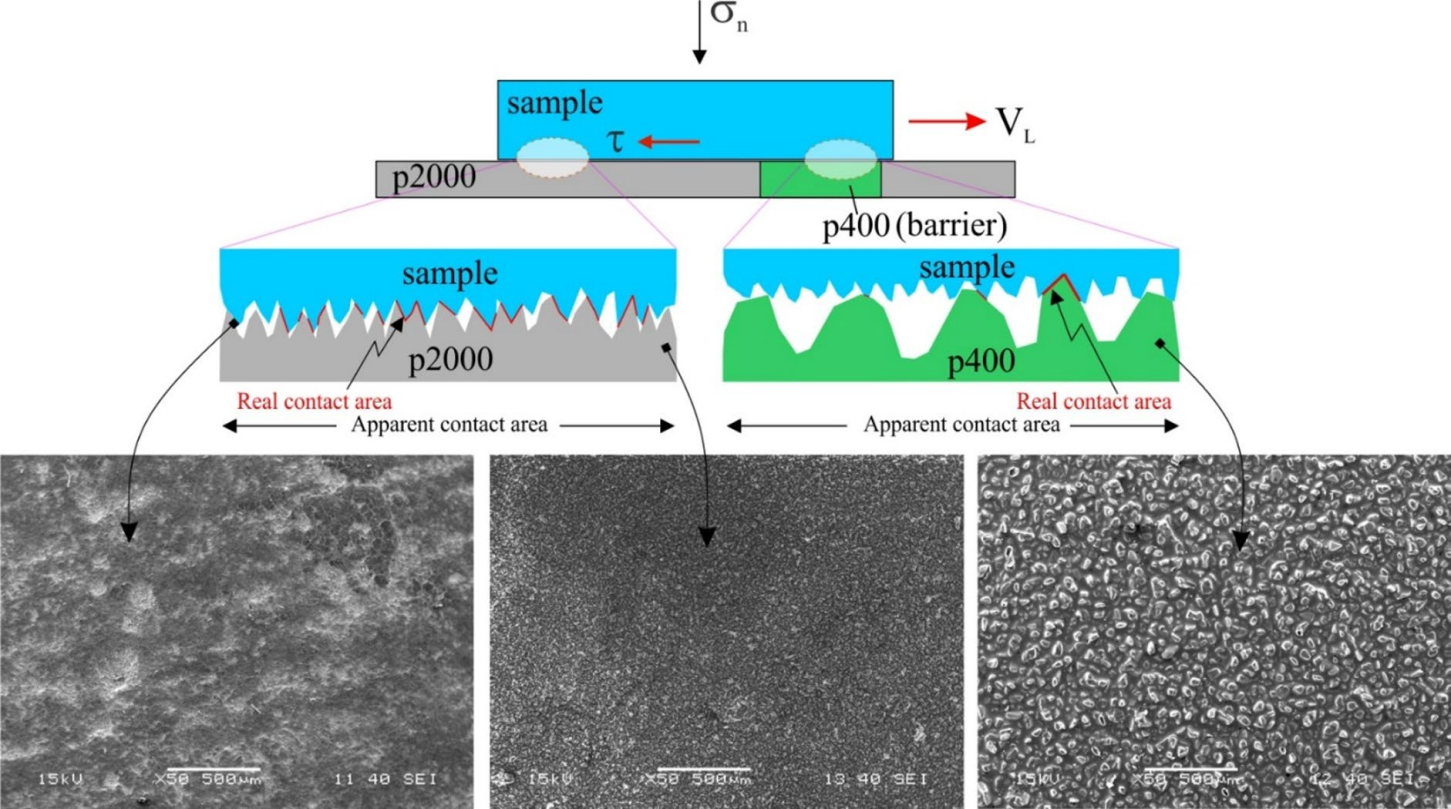
Schematic representation (side view) of contact between the sample (in blue) over frictionally heterogeneous surfaces (top panel, p2000 in grey and p400 in green). A zoomed-in schematic diagram shows (middle panel) the sample-p2000 surfaces (blue-grey) have more interlocking due to a greater real contact area (in red) than the sample-p400 interface (blue-green). The morphology images from Scanning Electron Microscopy of the Sample, p2000 and p400 sandpaper surfaces at 50X magnification (bottom panel). CorelDRAW (version 18; URL: https://www.coreldraw.com/en/) has been used to generate this figure.

**Fig. 3:**
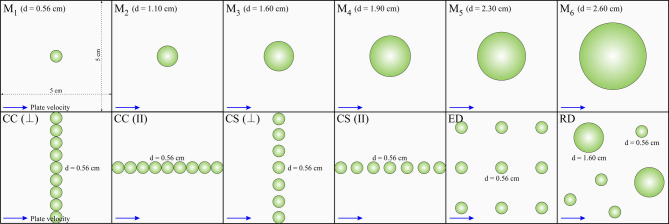
Schematic illustrations of the 2 types of experiments performed, named (top left corner in each setup) according to the geometry of barrier(green) introduced over the asperity (grey). The mean roughness parameter determines the frictional differences of asperity and barrier. The dimension of the sample placed over the frictional heterogeneity is 5 × 5 cm^2^. The loading velocity, analogous to the plate motion is indicated by the blue arrow. In the top panel, the diameter (d) of each single circular barrier (M_1_ to M_6_) increases from left to right. Multiple circular barrier (bottom panel) were introduced systematically. Terminologies and abbreviations are explained in Table 1. CorelDRAW (version 18; URL: https://www.coreldraw.com/en/) has been used to generate this figure.

Our laboratory experiments employ a single-degree-of-freedom spring-block system, where the sample connected to a force sensor, mounted in front of a linear slider, is moved over a frictional (homogeneous or heterogeneous) surface (5 × 5 cm base) under controlled loading velocity (2 μm/s). S-type force sensors (miniature load cells) can accurately measure both tension and compression using strain gauges, which detect material deformation as changes in electrical resistance. These are converted into voltage signals corresponding to the applied force. The experiments are characterised by alternating interseismic phases of stress build-up and followed by stress release. As the slider moves, compressive force builds up in the sensor (during the stick phase of the block) until it exceeds the static friction between the block and the surface, causing the block to slip. This cycle of force accumulation and sudden release produces the characteristic stick–slip motion. To simulate earthquake cycles over a frictionally heterogeneous surface, we used fine-grit p2000 sandpaper, which produced stick-slip behaviour analogous to locked asperities due to high interfacial coupling (Fig. 2), and coarse-grit p400 sandpaper, which promoted stable sliding akin to creeping barriers. Single circular barriers of varying sizes and multiple barriers with distinct geometries (e.g., linear clusters) were tested to assess their influence on slip dynamics. Force and acceleration data were recorded to classify seismic and aseismic events. Surface roughness characterisation (Fig. 2) and experimental configurations (Fig. 3), along with technical details, are provided in Materials and Methods and in the Supporting document (sections A and B).

**Table 1:** Summary of Laboratory experimental results with different types of barriers indicating area coverage and slip behaviour. The M_R_ is added as a reference, and note that the barrier area is 0, indicating that the fault area is entirely composed of the p2000 surface. It represents a smooth contact with the sample surface. M_7_ is another reference where the fault area is entirely composed of the p400 surface, note that the barrier area is 25 cm^2^.

Type of frictional heterogeneity (barrier)		Symbols	Experiment no.	Barrier area (cm^2^)	(Barrier area/apparent surface area)%	Slip behaviour
Reference frictional surface (without barrier)		M_R_	#236	0.00	0.00	Mega stick-slips
Single circular barrier		M_1_	#345	0.25	0.98	Relatively smaller stick-slips
		M_2_	#389	0.95	3.80	
		M_3_	#421	2.01	8.04	
		M_4_	#468	2.84	11.34	Aseismic Slips
		M_5_	#506	4.16	16.62	
		M_6_	#546	5.31	21.24	
		M_7_	#602	25.0	100	
Closely spaced linearly aligned multiple circular barrier		CC(⊥) CC(∥)	#742 #798	2.21	8.86	Smaller stick-slips and aseismic slips Aseismic slips
Far spaced linearly aligned multiple circular barrier (plate motion w.r.t linear)		CS(⊥) CS(∥)	#856 #910	1.72	6.89	Smaller stick-slips and aseismic slips
Evenly distributed multiple circular barrier		ED	#978	2.21	8.86	Rare stick-slips and mostly aseismic slips
Randomly distributed circular barrier		RD	#1010	4.18	16.74	Aseismic slips
Linear continuous barrier	Single	L	#1105–1365	2.8-4	11–16	Dominated by Aseismic slips (transient slow-slips and slow slips), except for orientation 45°
	Multiple	LS	#1415–1695	8.5–10	34–40	

#### Type I: Homogeneous fault surface (without frictional contrast)

**Fig. 4:**
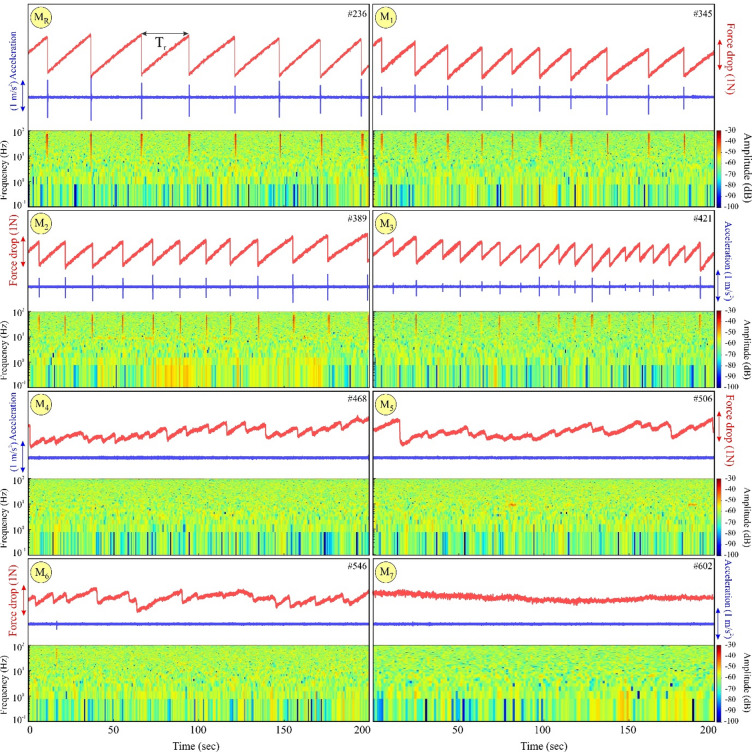
Results of single circular barrier stick-slip experiments. Force (in red) and acceleration (in blue) are recorded from the force sensor and accelerometer data, respectively, and their corresponding spectrogram of each experiment is plotted for 200s of experimental run time (note that M_R_ at the top left does not contain any barrier and is only used as a reference). In the spectrogram, high energy peaks (in the range of yellow to red) quantified by amplitude (analogous to higher seismic energy release) reaching frequency peaks of 10^2^ Hz are observed coinciding with the instantaneous slip phase (force drop and/or acceleration peaks), confirming that the observed slips are seismic in nature. The slip phases are isolated occurrences within the background noise, which is of lower amplitude (green). The isolated slip phases are interspersed between small amplitude (green and/or blue) lower frequency peaks (in the range of 1 Hz). T_r_ indicates the recurrence interval of slips. Note that stick slips vanish after M_3_. Python Script, Grapher graphical application (version 8.7.844; https://www.goldensoftware.com/products/grapher/) and CorelDRAW (version 18; URL: https://www.coreldraw.com/en/) have been used to generate this figure.

**Fig. 5:**
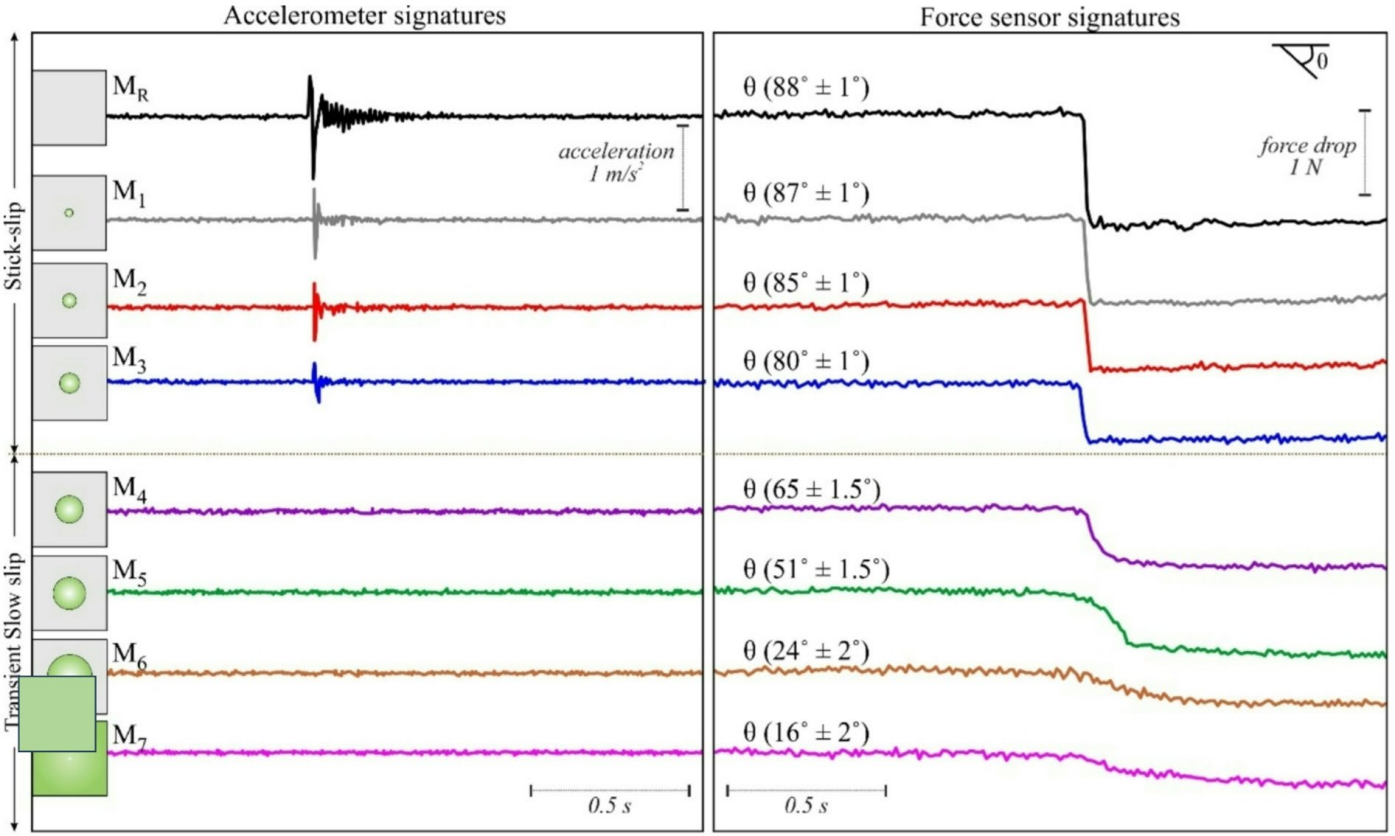
Acceleration (left panel) and Force drop (right panel) for 6 barriers (and M_R_, without barrier) are plotted for a time interval of 2 s, depicting the change in angle (θ) of slip and corresponding acceleration recorded in the accelerometer.

Analogue experiment performed on a frictionally homogeneous fault surface (that is, without frictional heterogeneity; similar to laboratory experiments of Ray et al.^90^), entirely covered by p2000 (Ra = 4 μm, Table S1) only, is taken as a reference (M_R_) for subsequent experiments (Fig. 4). 8 periodic stick-slip events are observed within the 200 s experimental run time. The slope of slip events is measured to be ~ 88º, and the slip duration is ~ 0.02s. The slope (θ, Fig. 5) of the slip phase is highest for this case. Consequently, the slip duration is lowest among all the subsequent cases with a frictionally heterogeneous fault surface (with barriers), suggesting that true stick slips (sudden drop in force) occurred for the reference experiment. The average recurrence time between each of the 8 periodic stick-slip events is 28s, and the force drop is 1.4 N. From the corresponding accelerometer data plot, it is observed that the peaks in acceleration coincide with the stick-slip events. The average acceleration observed is 1.3 m/s^2^. In the spectrogram, high-energy frequency peaks in the range of 10^2^ Hz are observed coinciding with the slips, confirming that the observed slips are seismic in nature. Another analogue experiment was performed, as a reference on a frictionally homogeneous fault surface (that is, without frictional heterogeneity), entirely covered by p400 (Ra ~ 10.56 μm, Table S1) only (M_7_). This experiment showed steady, stable sliding, as slip occurred without stick-slip or sudden failure (i.e., earthquakes), confirming the influence of the p400 surface.

#### Type II: Single circular barriers

To investigate the influence of discrete frictional heterogeneities on rupture dynamics and earthquake cycles, we conducted six distinct experiments (M_1_–M_6_) by sequentially introducing individual circular barriers into an otherwise homogeneous fault surface (from M_R_). Each experiment tested a unique barrier size while maintaining identical background frictional properties, allowing systematic analysis of scale-dependent barrier-rupture interactions. In Fig. 4, experiment M_R_ is used as a reference without any circular barrier. After adding a circular barrier M_1_ (area = 0.98% of the sample area, 25 cm^2^) at the centre of the asperity, the stick-slip events increased from 8 to 10 periodic slip events, with the average recurrence time (T_r_) being 17.53s compared to 27.8s in M_R_. The average force drop recorded is 1.27 N, which is 0.13 N less than that of M_R_. The average slope of 5 slip events is measured to be ~ 87º (Fig. 5), and the slip duration is 0.029 s. The slope decreased from M_R_ while the slip duration increased by 0.006s. In the accelerometer signal, acceleration peaks are observed corresponding to the slip events, similar to M_R_. The average amplitude recorded is 0.67 m/s^2^ (decreased by 0.61 m/s^2^ from M_R_). The amplitude of acceleration also decreased, as did the magnitude of force drop. In the spectrogram, high-energy frequency peaks in the range of 10^2^ Hz are observed, coinciding with the slip events and confirming that the observed slips are seismic.

Further increase in the barrier size (in M_3_, ~ 8% of the apparent contact area of the sample), 18 periodic stick-slip events are recorded in the same time interval of 200s. However, the periodicity of slip events is less consistent compared to the preceding cases. The average slope of slip events is ~ 80º (Fig. 5) and the slip duration is 0.09s. The slope decreased by 7º and the duration of slip increased by 0.06s compared to M_1_. The average recurrence time (T_r_) is 11s (6.53s less than M_1_). The magnitude of force drop varied moderately and so did the amplitude of acceleration, unlike M_1_ where it was more or less consistent throughout the recorded time. The average magnitude of force drop is 0.76 N (0.5 N less than M_1_), and the average amplitude of acceleration is 0.42 m/s^2^ (0.25 m/s^2^ less than in M_1_). The peaks in the spectrogram are of different amplitudes, brighter peaks with high amplitude corresponding to a large force drop is observed. All 18 slip events are seismic in nature, as confirmed from their signature in the spectrogram.

From M_4_ to M_6_, the accelerometer signal and the spectrogram are clean, i.e. sans peak acceleration and high-frequency peaks, respectively, suggesting transient slow slips and aseismic slips. The average duration of slow stick-slip for M_4_ is 0.16s, which is 0.07s higher than in M_3_, while the slope decreased to 65º, corresponding to a force drop of ~ 0.7 N. The duration of slow slip for M_6_ is 0.48s, which is higher than in M_4_, while the slope decreased to ~ 24º, corresponding to a force drop of 0.43 N. The barrier size in terms of area percentage (area%) of the apparent contact area of the sample in M_4_ is ~ 11%. Therefore, the transition from seismic (M_3_) to fully aseismic slip (M_4_) occurs within the range, indicating a threshold area between 8% and 11%. Translating this area threshold into a geometric scaling parameter, we calculated the ratio of the barrier diameter (along strike length, L_b_) to the along strike length of the contact interface (L) (Fig. S5). Thus, L_b_/L is within 0.32 (M_3_) to 0.38 (M_4_).

**Fig. 6:**
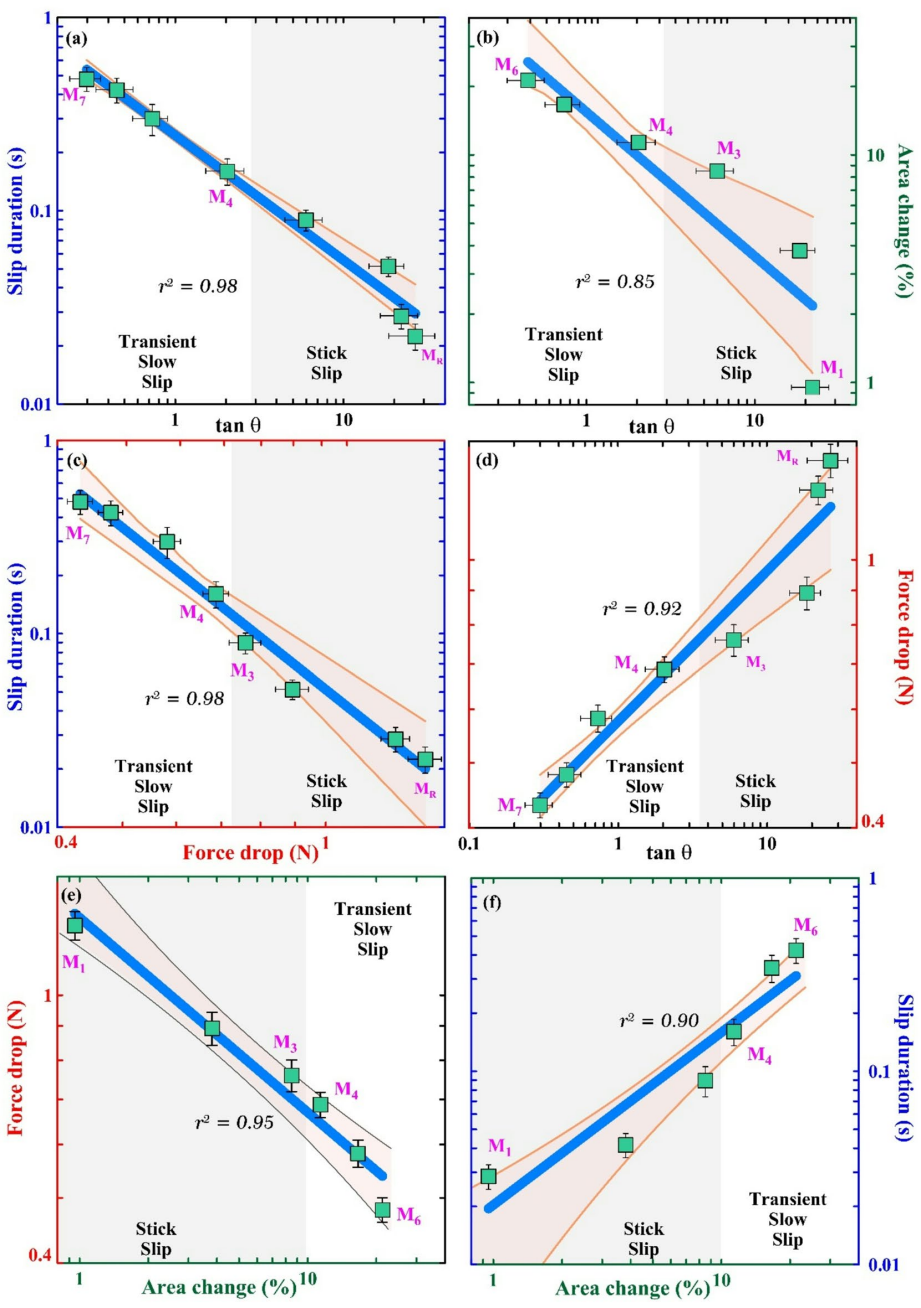
Graphs illustrating pairwise correlations between four key parameters: area percentage of the barrier with respect to the sample surface area (area%), force drop, slip duration, and *tan θ*. Each plot highlights the relationship between two variables, revealing consistent trends and interdependencies. Seismic stick slip (grey shaded region represents stick slip phase) is observed in M_1_ to M_3,_ followed by aseismic slip for M_4_ to M_7_. Note that the axes are in log-log scale, and figures containing Area change (%) only have 6 points because M_R_ (area change is 0, since it is without any barrier) and M_7_ (area change is 100%, since all of the fault interface is composed of p400 sandpaper) are used as references. The shaded region (light orange) represents the 95% confidence interval bounded by upper and lower limits (orange lines), indicating the range within which the true mean is expected to lie with 95% certainty. Stick-slip cycle recurrence time is highest for M_1_ (diameter ~ 0.56 cm) and lowest for M_3_ (diameter ~ 1.60 cm). Therefore, the recurrence time decreases with increasing area of the barrier. (**a**) Slip duration and tan θ are related inversely, and stick-slip behaviour exists till slip duration ~ 0.09s. With higher slip duration, the stick-slip character disappears. (**b**) Negative correlation exists between Percentage area and tan θ, stick slip behaviour disappears for tan θ < 6 or θ < 80.5º and area% > 8 (M_3_). (**c**) Slip duration decreases with an increase in force drop; force drop less than 0.76 N yields aseismic/slow slip behaviour. (**d**) Force drop and tan θ are found to be directly proportional, and M_R,_ which has the highest force drop, also has the highest slope (87.85º). (**e**) Negative correlation exists between force drop and percentage area covered by barrier (area%); for example, M_1,_ with the lowest area%, has the highest force drop. (**f**) Slip duration and area% are directly related to each other, i.e., slip duration is highest for M_7_ and gradually decreases for lower barrier size.

To summarise, the force drop decreases, and the slip duration increases as the barrier size is increased (as observed in M_1_ to M_3_). The fast stick-slip events persist till M_3_, beyond which a transition occurs from fast stick-slip to transient slow-slip and eventually to aseismic slip (M_4_ to M_6_). Along with the observable accelerometer signals and spectrogram signatures, the duration of slip is a key parameter to differentiate seismic fast slips or aseismic (transient slow-slip and slow-slip) slips. Slip duration and slope (tan θ) of slip events are related inversely (Fig. 6a). As a consequence of increasing area of the barrier, slip duration increases and the slope tan θ decreases, as is evident from the positive correlation with slip duration and a negative correlation with the slope (Fig. 6b and f). Stick-slip behaviour disappears for tan θ < 6 or θ < 80.5º and area % > 8 (M_3_) (Fig. 6b). Slip duration decreases with an increase in force drop. A Force drop < 0.76 N results in aseismic or slow slip (Fig. 6c). Force drop and tan θ exhibit a direct proportionality (Fig. 6d). For instance, experiment M_R_ which has the highest force drop also shows the steepest slope (~ 88º, Fig. 5). Finally, a negative correlation exists between force drop and percentage area (area%) covered by the circular barrier; for example, M_1_ with lowest area% exhibits highest force drop (Fig. 6e).

#### Type III: Multiple circular barriers

**Fig. 7:**
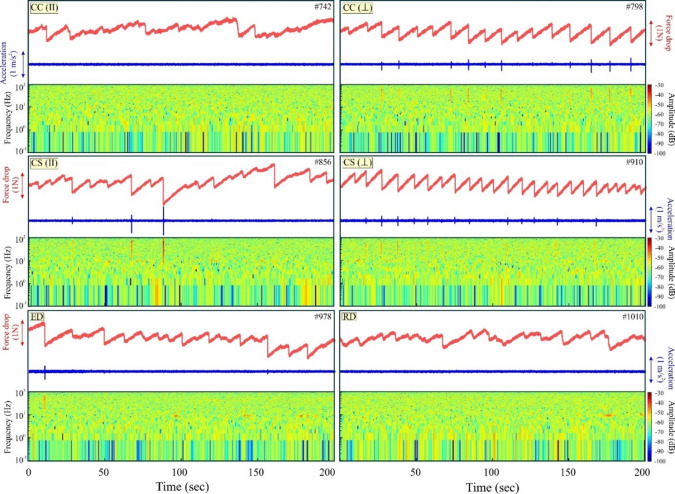
Results of multiple circular barrier stick-slip experiments. Force (in red) and acceleration (in blue) are recorded from the force sensor and accelerometer data, respectively, and their corresponding spectrogram of each experiment is plotted for 200s of experimental run time. The experiment numbers are mentioned in the top right corner of each plot. Note that stick-slip is absent in CC(II) and RD. Python Script, Grapher graphical application (version 8.7.844; https://www.goldensoftware.com/products/grapher/) and CorelDRAW (version 18; URL: https://www.coreldraw.com/en/) have been used to generate this figure.

The second set of experiments investigated the behavior of stick-slip cycles in the presence of multiple circular barriers arranged in different configurations (Fig. 3). In the CC(∥) configuration (barriers aligned parallel to the plate motion), no seismic slip was detected, as indicated by the absence of acceleration peaks and corroborated by the spectrogram (Fig. 7). Four prominent slip events were identified, characterised by slopes of less than 60º and slip durations exceeding 0.20s. In contrast, the CC(⊥) configuration (barriers aligned perpendicular to the plate motion) exhibited stick-slip events, which included both seismic and aseismic occurrences. Aseismic slips were identified by the absence of acceleration peaks and the lack of corresponding spectrogram signatures, whereas 10 seismic events were confirmed through the presence of acceleration peaks and high-frequency bright lines in the spectrogram. In the experiment CS(∥), three seismic slip events were recorded, while the majority of slip events were aseismic. For CS(⊥), periodic stick-slip (fast and transient slow-slip) events were observed. Among the 23 stick-slip events, 12 were identified as seismic, confirmed by distinct acceleration peaks and bright, high-frequency lines in the spectrogram plots. The aseismic slip events had a slip duration exceeding 0.16s in the CS cases as well. The distributed barriers only produced aseismic slips with rare seismic slips hosted by RD.

#### Type IV: Single and multiple linear continuous barrier

Further, a set of experiments was performed to investigate the impact of continuous linear barriers—akin to natural aseismic ridges as frictional heterogeneity—on the stick-slip cycle, considering both oblique and perpendicular convergence geometries (Fig. S6). All eight experiments exhibited erratic, non-periodic slip behaviour dominated by transient slow-slips and aseismic slip. Fast stick-slips were consistently absent, except in two experiment types (L_45°_ and LS_45°_), where the loading velocity is 45° to the barrier geometry. In these cases, occasional fast stick-slips were observed, indicated by peak accelerations and high-frequency signatures in the spectrogram.

#### Overview of experimental findings

Some key observations from the laboratory experiments can be summarised as follows: the area covered by barriers within an asperity primarily governs the onset of aseismic behaviour. In this context, the lower interseismic coupling at the barrier-sample interface compared to the asperity-sample interface plays a crucial role. From the single circular barrier experiments, stick–slip behavior is observed for barrier area ~ 8% (M_3_), while purely aseismic slip occurs at 11% (M_4_). Thus, the transition between these regimes lies within the range ~ 8–11%. Consequently, when L_b_/L exceeds ~ 0.38, fast stick-slip is absent and transient slow-slip or aseismic slip dominates. For multiple barriers, motion perpendicular to the linear geometry predominantly results in seismic behaviour, while motion parallel to it leads to predominantly aseismic slips. In the CC configuration, where area% is slightly higher than M_3_, both seismic and aseismic slips were observed, underscoring the significance of the motion’s direction. In contrast, the CS configuration, with a lower area% than M_3_ and CC, still exhibited seismic slips, emphasising the impact of barrier distribution even within a linear geometry. For the ED and RD configurations, which have area% values similar to CC and M_5_, mostly aseismic slips were observed. This further highlights the importance of barrier distribution, which, along with the geometry of motion, takes precedence over area% in determining seismic or aseismic behavior. This study examines a distinct phase of ridge influence: the frictional behaviour of fully subducted barriers. Our analogue experiments with Multiple Circular barriers (CC and CS) of linear geometry demonstrate that barrier geometry alone—parallel (||) or perpendicular (⊥) to the plate motion—governs slip modes without buoyancy effects. Subducted barriers aligned parallel to the plate motion (CC(||)) (i.e. perpendicular to the trench) eliminate seismic slip entirely, while barriers perpendicular to the plate motion (CC(⊥)) (i.e. parallel to the trench) enable mixed seismic-aseismic events to occur. In case of continuous barriers, oblique converges (L_45°_ and LS_45°_) occasionally produce smaller stick-slip events. Natural systems are more complex than idealised experimental configurations; numerous studies highlight the influence of subducting bathymetric features on megathrust rupture characteristics. For example, the Juan Fernández Ridge (JFR), Challenger Fracture Zone (CFZ), Chile Rise, Nazca Ridge, Medaña Fracture Zone, and Carnegie Ridge, along the Chilean and broader South American margin are known to act as persistent rupture barriers, impeding along-strike rupture propagation and defining major megathrust segments^51,91,92^.

### Numerical simulation of rupture propagation and stress evolution on a megathrust with heterogeneous frictional properties

The dimensionless parameter L_b_/L, which characterises the along-strike heterogeneity distribution, governs the transition between rapid stick-slip instabilities and aseismic slip transients. Our laboratory analysis demonstrates that synchronous ruptures of asperities across the barrier require L_b_/L to remain below a critical threshold value (~ 0.40). Such along-strike segmentation has been of critical focus in understanding rupture complexity. Previous studies have shown that lateral variations in fault frictional properties, such as alternating velocity-weakening and velocity-strengthening patches, can lead to rupture nucleation in isolated segments, rupture arrest at segment boundaries, or cascading rupture across multiple segments, depending on their strength contrast and separation distance^93,94^. These simulations underscore the importance of incorporating along-strike heterogeneity to realistically capture the rupture behaviour observed in natural faults. Building upon this discussion and our results from the previous section, we performed several quasi-dynamic simulations of earthquake sequences on megathrust and collisional settings with lateral variations in frictional properties, considering a rate-and-state-dependent friction aging law^95^. In the rate-and-state friction framework, fault segments that creep aseismically can be represented by VS material (a–b > 0), whereas locked segments are VW (a–b < 0). This simulation allows us to compute the long-term histories of fault slip on a simulated fault (schematic, Fig. S7), including rupture propagation and directivity. Three fault models were developed: A synthetic segmented fault with alternating VW/VS patches. The Alaska-Aleutian subduction zone (Shumagin Gap as a VS barrier). The Himalayan megathrust (DHR, MSR, and FR ridges as VS patches). Simulations spanned 500–1000 years to assess rupture dynamics across barriers. A detailed methodology is presented in the Materials and Methods section. The fault properties and geometries for the three simulations are described in the following sections and Table S2 of the Supporting document.

#### Synthetic fault simulation with alternating VW and VS patches

**Fig. 8:**
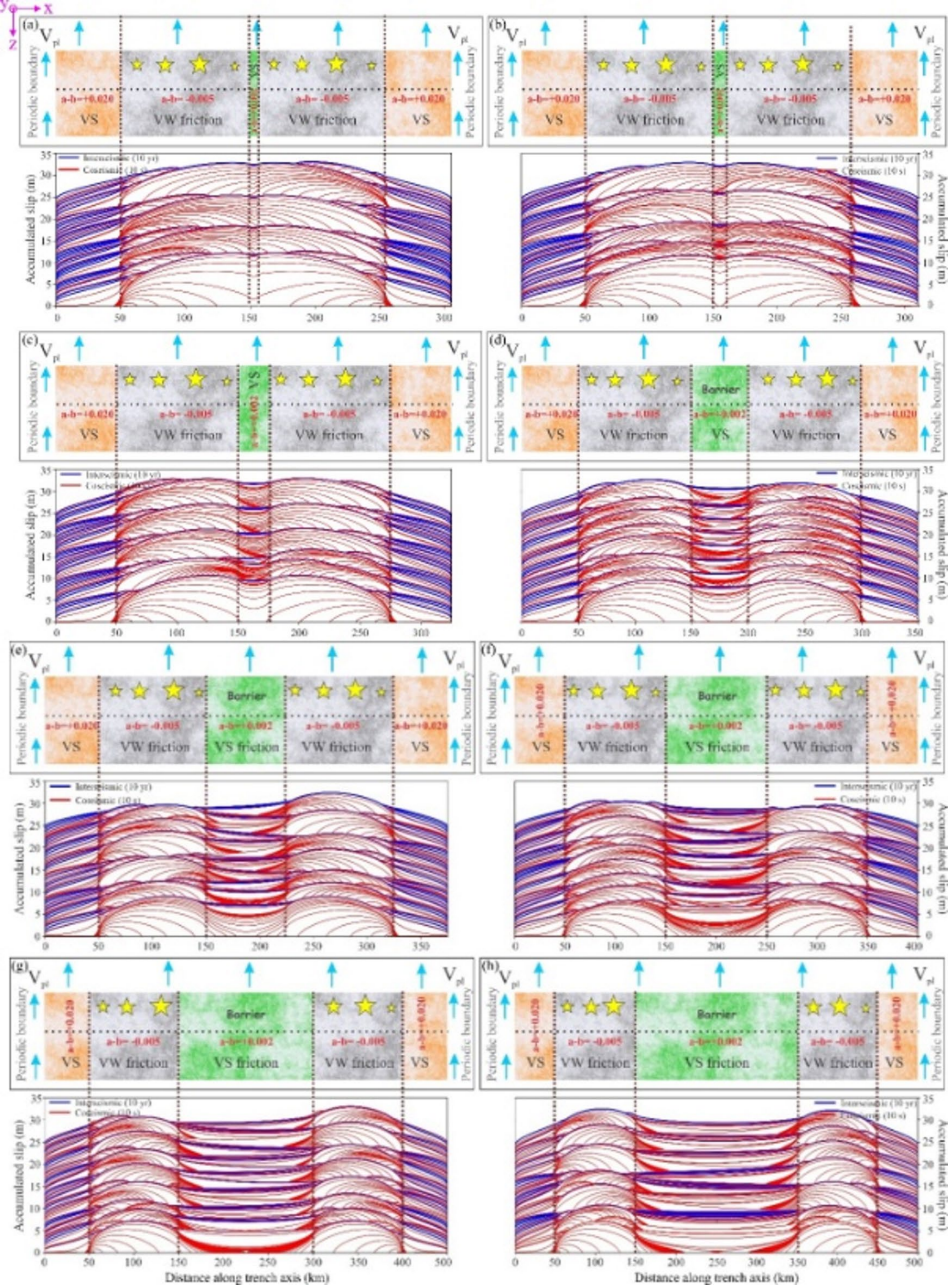
(**a** to **h**) Variation of frictional parameter (**a**–**b**) along the strike of an idealised trench. It is bounded by buffer zones on either side. The velocity strengthening (VS) zone in the middle is bounded by velocity weakening (VW) zones on either side. The width of the VS zone is increased subsequently as follows: 5 (**a**), 10 (**b**), 25 (**c**), 50 (**d**), 75 (**e**), 100 (**f**), 150 (**g**), 200 km (**h**) (top panels). Contours of cumulative slip accumulation on the fault over a 500-year quasi-dynamic simulation, incorporated with rate-and-state friction law. The red lines represent slip accumulation every 10 s during the simulated seismic events when the maximum slip velocity exceeds 1 mm s^− 1^. The blue lines represent slip accumulation every 10 years, which shows the aseismic behaviour of the fault (bottom panels). CorelDRAW (version 18; URL: https://www.coreldraw.com/en/) and Quasi-DYNamic earthquake simulator (v1.1, URL: https://zenodo.org/records/322459) have been used to generate this figure.

We considered a model geometry with alternating frictional properties (i.e. VS and VW), loaded by a constant plate motion (V_pl_ = 50 mm/yr). The model parameters and the model geometry are presented in supplementary Table S2 and Fig. 8, respectively. The histories of slip for 2D QDYN simulation shows the accumulation of slip during the interseimsic periods is represented by the blue lines indicating aseismic behaviour while slip during coseismic phase is shown in red (Fig. 8). We systematically increase the along-strike length of the VS patch, keeping the length of the VW segments on either side of it, constant. Note that the length of the fault segment is simultaneously increased to accommodate this adjustment. The VS patches on either side of the model (buffer zones) act as permanent barriers. The slip rate at VS areas is comparable to plate velocity, while the VW regions remain almost fully locked during interseismic periods. First, the length of the central VS segment is small (5 km), and the majority of the earthquake ruptures nucleating in one VW region easily propagate through the VS region, indicating stress transfer and partial or full rupture of the adjacent VW region. As the length of the central VS segment is increased, higher is the average stress increment required for seismic slip within the VS patch, hence we get an increasingly lesser number of through-going earthquake ruptures. We note that the VS patch acts as a permanent barrier, stopping majority of the through-going coseismic rupture once it reaches a characteristic length (150 km, Fig. 8g). The L_b_/L is ~ 0.43 in this case. Earthquakes nucleating in the VW region mostly fail to cross the VS patch and terminate at the rheological transition from VW to VS. Moreover, the earthquakes that nucleate in the rheological transitions from VS to VW regions and terminate within the VS region.

#### Shumagin Seismic gap in the Alaska-Aleutian subduction zone

**Fig. 9:**
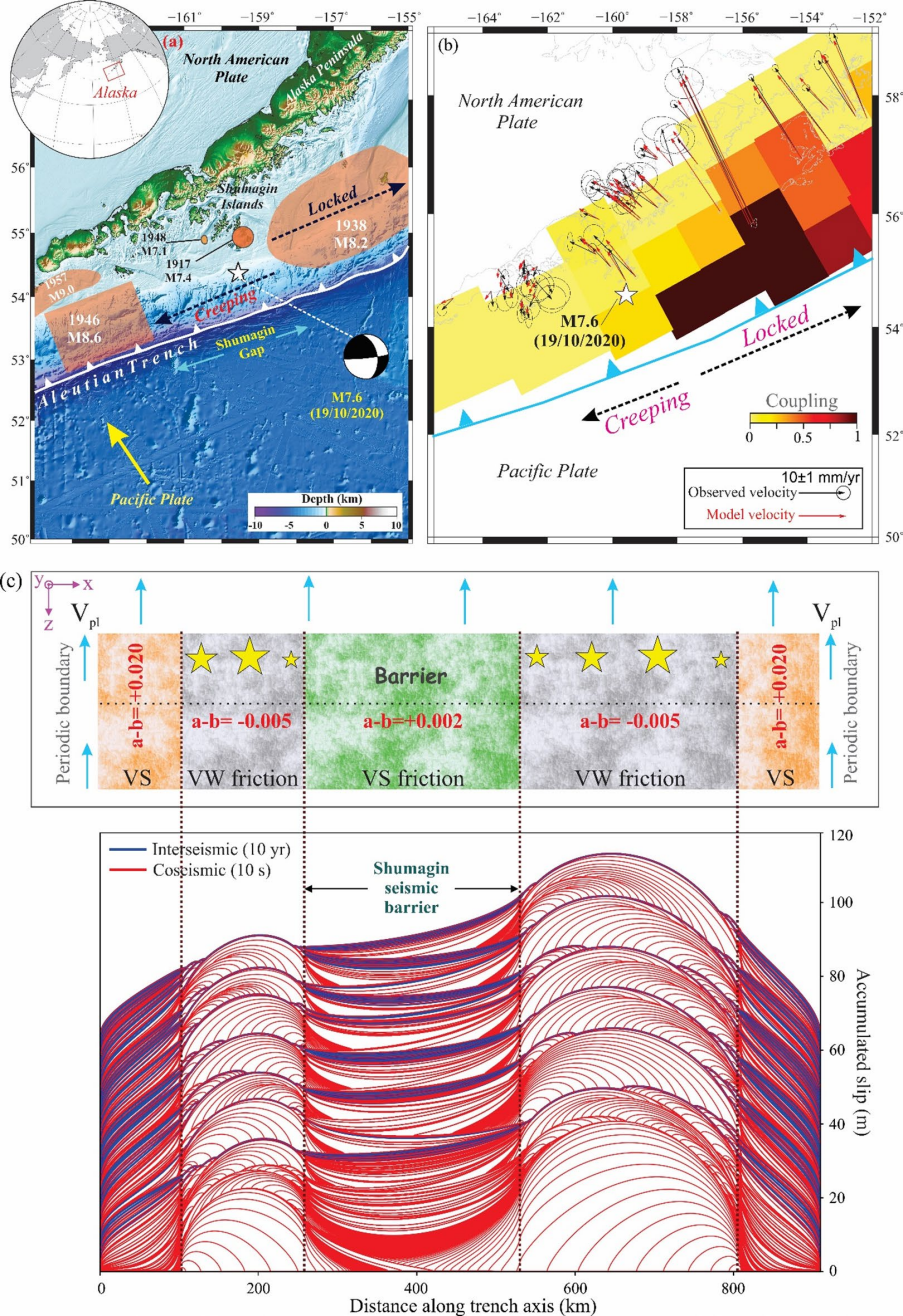
(**a**) Tectonic setting and bathymetry of the Aleutian trench (fault line marked by white line with triangles) along with the epicentre of the *M*w 7.6, 2020 earthquake. Note that the location of the *M*w 7.6 earthquake is along the transition zone between the locked and creeping segments. The light red areas indicate rupture zones for the great historical earthquakes. Inset with red box marks the study area considered for the analysis. (**b**) Represents the interseismic coupling map (modified after Senapati et al.^102^) obtained from the inversion of the horizontal GNSS velocity fields along the Aleutian trench with respect to the North American plate, considering a smoothing parameter on the chi-square criteria. Zero represents no coupling along the plate interface, while one corresponds to the complete coupling between the two plates. The modelled and observed horizontal GNSS velocities along the trench are indicated by the red and black vectors. The grey dashed line marks the transition between the locked and creeping segments. (**c**) Top panel: variation of the friction parameter (*a–b*) along the strike of the trench. The fault is divided into different segments based on the frictional parameter (*a–b*) (i.e. VW and VS zones). The 270 km velocity strengthening zone is bounded by 160 km (left) and 290 km (right) velocity weakening zones and a 100 km VS buffer zone on the left- and right-hand sides, respectively. Bottom panel: contours of cumulative slip accumulation on the fault over a 1000-year quasi-dynamic simulation, incorporated with rate-and-state friction law. The red lines represent slip accumulation every 10 s during the simulated seismic events when the maximum slip velocity exceeds 1 mm s^− 1^. The blue lines represent slip accumulation every 10 years, which shows the aseismic behaviour of the fault. The figures were generated using Generic Mapping Tools (version 5.2.1; URL: https://www.soest.hawaii.edu/gmt/), CorelDRAW (version 18; URL: https://www.coreldraw.com/en/) and Quasi-DYNamic earthquake simulator (v1.1, URL: https://zenodo.org/records/322459).

The Shumagin Seismic Gap (i.e. from 162°W–158.5°W, Fig. 9a) of the Alaska-Aleutian subduction zone is characterised by notably low seismic coupling, indicating that a significant portion of plate convergence is accommodated through aseismic slip rather than large megathrust earthquakes^75,96^. In contrast, regions adjacent to the gap, such as the Semidi segment to the east and the Unalaska segment to the west, exhibit higher seismic coupling, with historical records of large earthquakes suggesting a greater degree of strain accumulation and release^97,98^. To quantify along-strike variation in megathrust coupling and associated strain accumulation at the Shumagin seismic gap, we have analysed geodetic data sets covering the entire span of the Alaska–Aleutian subduction zone, compiled from several previous studies^97,99–101^. The interseismic coupling model (Fig. 9b) was derived through a sensitivity analysis where fault-patch boundaries in the inversion were systematically shifted along-strike^102^. Despite these variations, the models consistently resolved a sharp transition near the Shumagin Islands, with the eastern segment (Semidi region) showing robust, strong coupling. The western segment (Unimak region) exhibits lower modeled coupling, which may partly reflect limited offshore geodetic resolution. Therefore, for our quasi-dynamic simulation, we represent the along-strike segmentation using scaled lengths based on the estimated rupture extents of the bounding great earthquakes^102,103^: the central velocity-strengthening (VS) zone (270 km) represents the Shumagin gap, bounded by velocity-weakening (VW) zones approximating the 1946 M 8.6 rupture to the west (160 km) and the 1938 M 8.2 rupture to the east (290 km). The details of the procedure are adapted from Senapati et al.^102^. The coupling map (Fig. 9b) indicates the heterogeneous coupling within this section of the subduction zone, where zero represents the regions with aseismic deformation/slip and one represents fully locked segments.

In Fig. 9c, we examine a model configuration consisting of alternating frictional domains with velocity-strengthening (VS) and velocity-weakening (VW) behaviour, driven by the maximum reported long-term convergence rate of the Pacific Plate (V = 57 mm yr⁻¹)^101,104^. The adopted model geometry and parameters are summarized in Fig. 9 and Table S2. Although the setup assumes a simplified two-dimensional geometry and a binary distribution of frictional properties, the simulations generate earthquake cycles that resemble natural behaviour over 1000 years (Fig. 9). In this framework, the VW patches are the primary sites of earthquake generation, producing events with variable rupture sizes and propagation directions. In contrast, the VS regions predominantly accommodate slip aseismically and behave as persistent barriers to rupture propagation. Nevertheless, short-term interactions between the two VW segments occasionally occur, as indicated by events lacking or showing only limited blue interseismic slip contours between them.

Rupture behaviour near the frictional transition between VW and VS regions is particularly complex. In several cases, earthquake ruptures extend partially into the nominally VS domain (Fig. 9c), suggesting that coseismic slip can locally propagate into areas that otherwise exhibit velocity-strengthening behaviour. Despite this complexity, the model indicates that a VS zone with a width comparable to that of the Shumagin seismic gap can effectively prevent through-going ruptures, although earthquakes occurring within the adjacent VW regions may still interact over timescales of several years. For this configuration, the ratio ${\mathrm{L}}_{\mathrm{b}}/\mathrm{L}$ is ~ 0.38. If the extent of the VS region (L_b_) gets diminished with respect to the total fault length, over a geological timescale due to plate convergence and subduction, VW zones may interact by synchronous or through-going ruptures crossing the VS region in the middle. This is further evidenced by the results of Fig. 8a–f, where the barrier effect is only prominent for L_b_/L ~ 0.43.

#### Himalayan deformation front and segmentation

**Fig. 10:**
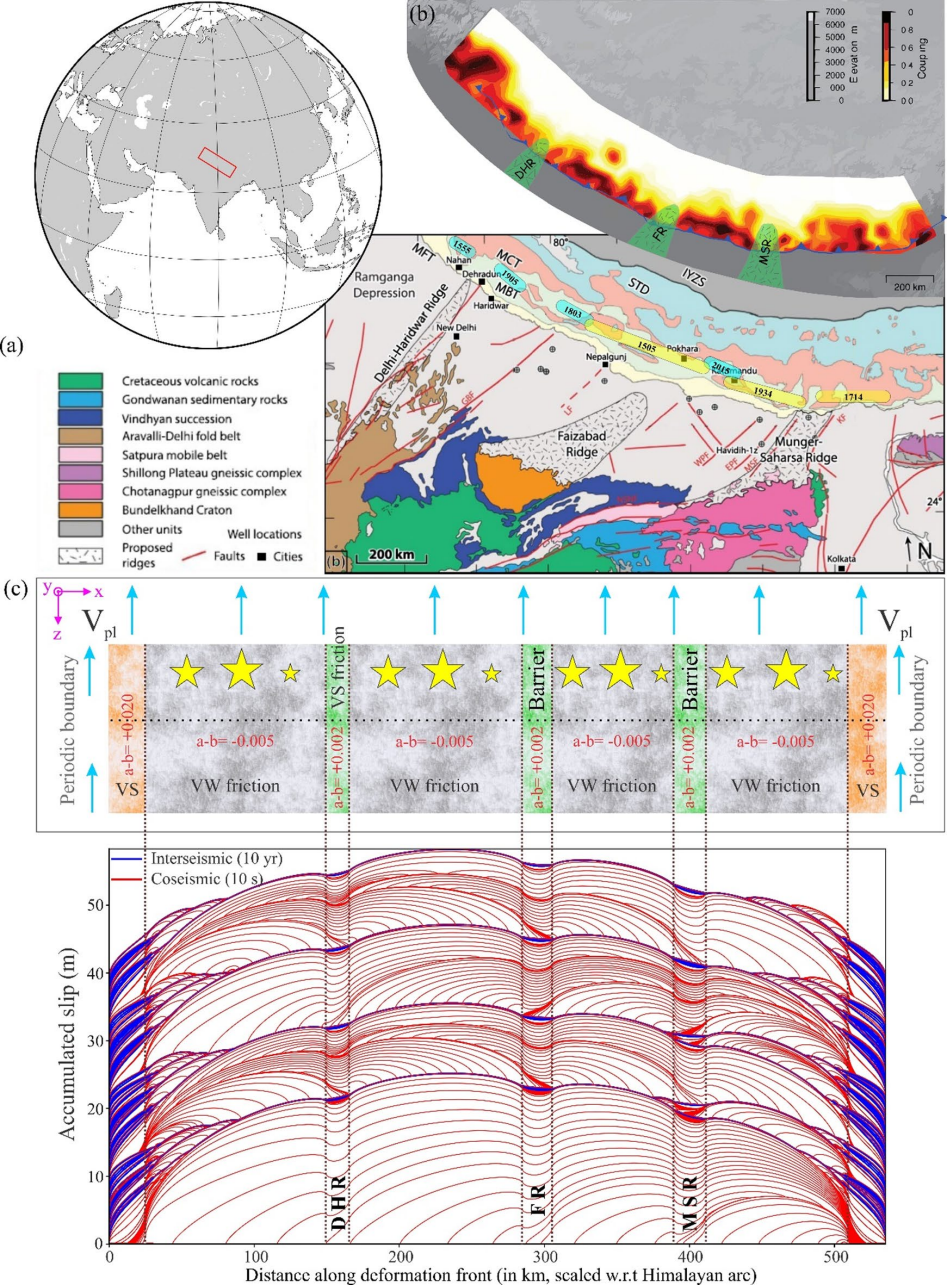
Regional political map of south-central Asia. (**a**) Generalized geology map of Northern India, Nepal, and adjacent areas (modified after Duvall et al.^150^ and references therein), and United States Geological Survey public data. Approximate traces of basement ridges after Godin and Harris^151^. IYZS: Indus–Yarlung Zangbo Suture Zone; STD: South Tibet Detachment system; MCT: Main Central Thrust; MBT: Main Boundary Thrust; MFT: Main Frontal Thrust; KF: Kishangang Fault; MSF Munger-Saharsa Ridge Fault, WPF West Patna Fault; EPF: East Patna Fault; LF: Lucknow Fault; GBF: Great Boundary Fault; NSNF: North Son-Narmada Fault; Schematic representation of rupture areas (yellow, rupture that reached the surface and blue, subsurface rupture) and their along-arc extent estimates of great earthquakes (M > 7.5) and larger events in the Himalayas since 1500 CE (modified after Zilio et al.^73^ and references therein). (**b**) Present-day topography and coupling distribution inferred from the posterior mean-coupling model. Solid blue line shows the surface trace of the Main Frontal Thrust. Schematic representation (in green) of the positions of the three subsurface ridges. (**c**) Variation of the friction parameter (a–b) along strike of the Himalaya. The fault is divided into different segments based on the friction parameter (a–b) (i.e., VW and VS zone). The different ridges are considered as VS zones and a 100 km velocity strengthening buffer zone on the left and right sides, respectively (top panel). Contours of cumulative slip accumulation on the fault over 500 years’ quasi-dynamic simulation, incorporated with rate-and-state friction law. The red lines represent slip accumulation every 10s during the simulated seismic events when the maximum slip velocity exceeds 1 mm/s. The blue lines represent slip accumulation every 10 years, which shows the aseismic behaviour of the fault (bottom panel). CorelDRAW (version 18; URL: https://www.coreldraw.com/en/) and Quasi-DYNamic earthquake simulator (v1.1, URL: https://zenodo.org/records/322459) have been used to generate this figure.

The Himalayan megathrust exhibits spatial variability in interseismic coupling, segmented by low-coupling regions along strike (Fig. 10a and b), that align with three subsurface Indian basement ridges: The Delhi-Haridwar Ridge (DHR), Faizabad Ridge (FR), and Munger-Saharsa Ridge (MSR), suggesting segmentation of the Main Himalayan Thrust (MHT) (Zilio et al., 2020). Despite significant uncertainties, rupture extents of major Himalayan earthquakes over the past millennium correlate with ridge subduction and coupling patterns, as ruptures do not cross segment boundaries defined by lower-plate structures^105,106^. For example, the 1905 Kangra rupture’s eastern edge coincides with the DHR^27,107^, the 1803 rupture abuts the DHR^108^, and the 1934 Nepal-Bihar rupture reached the MSR^109^. However, rupture extent estimates and ridge extrapolations may have an error of ~ 50 km^27^.

To investigate the role of these ridges in earthquake rupture dynamics, numerical modelling has been employed. Such a model simulates rupture propagation and stress evolution along the Himalayan megathrust, considering the interaction of different ridges and varying frictional properties and helps to understand how features like the DHR, FR, and MSR influence earthquake behaviour, particularly in terms of rupture directivity and event size. We considered a model geometry with alternating frictional properties (i.e., VS vs. VW), loaded by the maximum reported long-term plate motion of the India plate (V_L_ = 40 mm/yr) (Fig. 10c). The lengths of the deformation front, including the four VW zones and segmented by the three subsurface ridges, are scaled relative to their natural dimensions, and the simulation is run for 500 years to minimize computational time. The analysis reveals fault patches with VW frictional behaviour are rich in earthquake production, with varying rupture directivity and event size. The predominantly creeping patches characterised by VS frictional behaviour are stable in nature. From the simulation result, it has been observed that the subsurface ridges in the Himalayas (DHR, MSR, FR) do not act as absolute or permanent barriers to earthquake rupture propagation. The scaled geometry reveals that their along-strike extents of each ridge, are significantly smaller than the total Himalayan megathrust segment that we have considered, placing their Lb/L ratio < < 0.38 threshold established earlier for a barrier to act as a permanent barrier. Instead, they function as conditional barriers, where dynamic ruptures can frequently bypass them given sufficient stress accumulation and favorable fault rheology (Fig. 10).

## Discussions

Laboratory investigations of rock friction demonstrate that fault surface morphology exerts a strong control on slip stability. Experimental studies show that comparatively smooth faults tend to transition from stable sliding to stick–slip at higher normal stresses than rough faults, highlighting the influence of surface roughness on displacement-driven fault slip behaviour and frictional response^110–113^. These studies further suggest that fault stability reflects a system-level response governed by the real contact area between fault surfaces and the elastic stiffness of the surrounding medium. Smooth faults generally develop more continuous contact and therefore a more homogeneous stress field during stick–slip cycles, whereas rough faults promote stronger spatial stress heterogeneity. Such differences in stress distribution may favour the occurrence of larger stick–slip events on smoother faults^114^. According to the asperity model, Chile-type zones^115^ are characterised by simple broad contact interfaces with uniform stress levels and strong coupling. Our laboratory experiment without a VS patch/barrier (M_R_) indicates similar characteristics with quasi-periodic stick-slips which are assumed to be analogue for large earthquakes. Such quasi-periodic nature persists, even after the introduction of single circular barriers, although the stick-slips become more frequent with smaller magnitude and less energy release until the area of the barrier with respect to the interface area is ~ 11%. Beyond this threshold area%, fast stick slips are absent, and slip becomes aperiodic with transient slowslip and aseismic slip dominating. Many natural faults are believed to consist of VW patches surrounded by VS sections. Song and McLaskey^116^ investigated how the spatial distribution of VS and VW regions influences fault slip behaviour. Their laboratory experiments simulate natural faults using plexiglass samples to represent VW regions, with Teflon-coated sections acting as VS barriers that contain ruptures within the VW areas. The findings reveal that asperity size (quantified by the ratio of asperity length, L and the critical nucleation length, $${h}^{*}=\raisebox{1ex}{$G$}\!\left/\!\raisebox{-1ex}{${K}_{c}$}\right.$$, where $$G$$ is the shear modulus and $${K}_{c}$$ is the critical stiffness) directly impacts rupture dynamics, with larger asperities driving a transition from aseismic slip to periodic stick-slip, and eventually to non-periodic stick-slip behaviour. Conversely, our experimental results indicate the effect of barrier size (governed by area) on fault slip behaviour such that increasing the area% covered by the barrier (or decrease in the asperity area) results in a transition from fast stick-slips to a combination of transient slow-slips and aseismic slips to ultimately slow slips. These results highlight the critical role of fault heterogeneity in earthquake mechanics. Models characterised by a very rough interface have lower integrated fault strength and lower interseismic coupling than models with a smooth interface^57^. Our experiments with barriers represent lower interseismic coupling and show aperiodic stick-slips with a combination of transient slow slips and aseismic slips (CC and CS experiments).

A key aspect of our laboratory experiments is the generation of a range of slip behaviors: fast stick-slips, transient slow-slips and aseismic slips (the differences in naming conventions like slow slip, transient aseismic slip, afterslip, active fault or surface creep etc. likely originate from the different communities that have explored different geological contexts in parallel series of discoveries and advances). Leeman et al.^117^ have shown fast stick slips (~ 0.01s) transition to transient slow-slips (0.75s) under decreasing normal stress. Im et al.^118^ reported slow slip events of 1s. From our laboratory experiments, we report the duration of fast stick slips is in the range of > 0.03s (M_R_), the transient slow-slips had a duration of > 0.16s (M_4_, CC and CS) while the aseismic slip durations were usually > 0.75s (M_5_, M_6_, ED and RD).

A central problem in seismotectonics concerns whether earthquake ruptures can propagate across faults characterised by heterogeneous mechanical properties. Frictional heterogeneities are therefore considered an important factor that may limit the growth of large earthquakes, whereas ruptures can propagate more freely along relatively uniform fault interfaces. In particular, fault systems consisting of seismogenic patches separated by creeping barriers have attracted considerable attention, motivated by geophysical observations^62,64,66–68^. This conceptual framework is widely adopted in numerical studies of earthquake sequences, including investigations of repeating earthquakes^116^. The geometric partitioning of faults into seismic and aseismic zones control on rupture propagation and synchronised failure of asperities across barriers. Our experimental results demonstrate that this transition in slip behavior is systematically governed by along strike arrangement of barriers and asperity, defined by the critical threshold (~ 0.32–0.38) of the dimensionless parameter L_b_/L beyond which the adjacent asperities separated by the barrier will not fail together simultaneously i.e. the barrier will act as a permanent barrier to impede coseismic rupture propagation by promoting slip behaviour dominated by transient slow-slips and slow slips. This finding aligns with previous studies of Corbi et al.^69^, who, using 3D analogue modelling, demonstrated that the along-strike barrier-to-asperity length ratio controls asperity synchronization, identifying a transition to permanent barrier behaviour when the ratio exceeds 0.5. The similarity of our critical threshold (L_b_/L > 0.4) with this previous study, despite differences in methodology and scaling underscores the fundamental role of along-strike geometric partitioning. While the precise values may vary due to rheological and model-specific factors, both parameters capture the same underlying principle: the relative along-strike extent of a barrier determines its efficacy in stopping ruptures.

Megathrust faults, composed of multiple sub-segments in the seismogenic zone, can rupture individually or together with adjacent segments during larger seismic events. Their non-periodic or even chaotic behaviour results from stress transfer and its influence on prestress conditions. The synchronisation of adjacent asperities and overcoming intervening barriers can lead to the occurrence of larger seismic events and associated tsunamis. For example, the 1960 Mw 9.5 Chile and the 2004 Mw 9.2 Sumatra–Andaman earthquakes, where inversions of geological and geophysical data reveal the rupture of multiple (> 4) high-slip patches^69^. Similarly, analyses of seismic, geodetic, and tsunami observations indicate that the 2001 Mw 8.4 Arequipa (Camaná), Peru, earthquake involved the failure of two spatially offset asperities^92^. Geodetic and seismic observations reveal other natural analogues to this persistent barrier behavior, such as the Batu Islands segment of the Sunda megathrust, where a narrow low-interseismic-coupling zone separates more strongly locked patches, coincident with a relative absence of great ruptures^64^. Similarly, the 2007 Pisco earthquake (Mw 8.0) in Peru ruptured two locked patches but was arrested by an intervening velocity-strengthening barrier, underscoring how such zones can modulate or stop rupture propagation^65,66^. Interactions between neighbouring asperities have commonly been examined using simplified analytical representations such as coupled spring–slider models, where individual sliders represent adjacent fault segments. Even within this idealized framework, stress transfer between segments can generate considerable spatial and temporal variability in seismic behaviour^8,119^. Numerical modelling studies have further investigated the role of barriers by introducing prestressed heterogeneities or a frictional heterogeneity along the fault interface. These studies show that rupture propagation across such barriers depends strongly on the applied loading conditions and the mechanical characteristics of the heterogeneity, such that ruptures may either propagate through the barrier or arrest at it^24,120^. More sophisticated fully dynamic rupture simulations that incorporate rate-and-state friction and account for both coseismic and dynamic stress transfer provide additional insights into rupture evolution and its implications for seismic hazard assessment^65,89^. These models have been used to investigate the probability that a seismic rupture overcomes barriers, producing, in turn, a large magnitude earthquake.

Barriers may be persistent (rarely or never traversed), frequent (occasionally traversed), or ephemeral (changing location from cycle to cycle). With shorter historical records or imprecisely known rupture endpoints, it can be difficult to distinguish whether a barrier is frequent or ephemeral^121^. With continuous subduction, a transition from one type to another, for example, persistent to an ephemeral barrier, is possible within a segment. Our synthetic fault simulation demonstrates that as the size of the velocity-strengthening (VS) barrier separating adjacent asperities increases, it progressively transitions from a frequent to a persistent barrier. This transition occurs at a critical threshold, where the L_b_/L is approximately 0.43. This critical value is similar to our numerical observations of the Alaska-Aleutian subduction zone, where the Shumagin Gap, a well-documented seismic barrier, constitutes approximately 0.38 (L_b_) of the total fault length (L). This estimate considers the Unimak and Semidi segments to the west and east of the Shumagin Gap, respectively, as frictionally locked. Our laboratory experiments with a single circular barrier match with the numerical simulation results, particularly as the barrier in M_4_ (L_b_/L = 0.38) behaving as a persistent barrier marking the threshold beyond rupture propagation in the form of fast stick slip is inhibited and transient slow-slip to slow-slip behaviour dominates. The consistency of the L_b_/L threshold (~ 0.4) across our laboratory experiments and numerical simulations—including the Alaska-Aleutian subduction zone where the Shumagin Gap constitutes ~ 0.38 of the fault segment—validates this dimensionless ratio as a robust parameter that links simplified models to natural fault behaviour. Its empirical calibration across multiple approaches makes it a promising, observationally-constrained tool for estimating the barrier potential of fault segments. As Corbi et al.^69^ noted, such dimensionless ratios are particularly valuable because they can be constrained in nature through observed asperity–barrier distributions from interseismic coupling and coseismic slip patterns.

The along-strike rupture extents of great Himalayan earthquakes—such as the 1934, 1505, and 1255 events—remain debated due to sparse paleoseismic and historical constraints. While structural barriers like the Munger-Saharsa Ridge (MSR) and Faizabad Ridge (FR) are hypothesized to segment ruptures, their effectiveness as absolute barriers is uncertain. Paleoseismic evidence suggests the 1934 earthquake may have crossed the MSR^122^, but the absence of definitive surface ruptures near the ridge leaves this interpretation unresolved. Historical accounts of the 1505 earthquake describe widespread destruction from Kumaon to Kashmir^123^, yet whether the rupture breached the FR remains speculative due to limited trenching data. The 1255 event, documented primarily in Kathmandu chronicles, lacks sufficient evidence to confirm rupture beyond central Nepal^124^. Numerical simulations, such as those conducted with QDYN in this study, provide critical insights where geological and historical records are incomplete. Our results demonstrate that dynamic ruptures can propagate across the MSR and FR, contradicting the assumption of rigid segmentation. This aligns with dynamic rupture models^73^ showing that stress transfer and fault rheology may enable cascading ruptures. While paleoseismic studies offer snapshots of past events, numerical modelling explores physically plausible scenarios that may not yet have empirical corroboration.

### Concluding remarks

This study focuses on large earthquakes on subduction megathrusts, investigating the role of frictional and geometric heterogeneities in controlling seismic and aseismic slip behaviour. Through laboratory experiments and quasi-dynamic numerical simulations, we have explored the influence of velocity-strengthening barriers on earthquake cycles. The primary outcomes of the study are highlighted below:

1. From our laboratory experiments, we establish that frictionally heterogeneous fault surfaces (in terms of surface roughness) modulate characteristics of slip behaviour depending upon the area, geometry, and distribution of velocity-strengthening barriers. Increasing the area of a single circular barrier within an asperity leads to a transition from periodic stick-slip (seismic events) to aseismic slip when the barrier area exceeds ~ 11% of the apparent contact area of the sample. For multiple barriers, the orientation relative to the loading direction plays a crucial role, with perpendicular arrangements promoting seismic slip and parallel arrangements favouring aseismic behaviour.
2. Numerical simulations of a synthetic fault and the Alaska-Aleutian subduction zone reveal that a velocity-strengthening patch acts as a permanent rupture barrier when its length constitutes ~ 0.40 of the fault segment. For the Shumagin Gap, the observed L_b_/L of 0.38 falls precisely within—and aligns with the upper end of—the critical threshold range (0.32–0.38) identified in our laboratory experiments This consistency validates L_b_/L as a robust scaling parameter across methodologies.
3. For a different tectonic setting, such as the Himalayan deformation front, it is well known that the lateral extent of great Himalayan earthquakes remains uncertain due to (a) incomplete paleoseismic records across structural barriers, (b) ambiguities in historical accounts of pre-instrumental events, and (c) conflicting evidence regarding whether ridges like Munger-Saharsa and Faizabad fully inhibit rupture propagation. Our numerical modelling demonstrates that these ridges act as conditional barriers; they influence—but do not necessarily terminate—dynamic ruptures, suggesting historical earthquakes such as (the 1505, 1934 and 1255 earthquakes) may have propagated farther than geological evidence currently indicates.

The above findings underscore the significance of frictional heterogeneities in influencing earthquake rupture propagation and segmentation. Our results suggest that the along-strike length of velocity-strengthening patches has a profound effect on long-term seismic behaviour, providing insights into the persistence of seismic rupture segmentation and asperities. To facilitate comparison with natural subduction systems, we introduced the dimensionless parameter ${\mathrm{L}}_{\mathrm{b}}/\mathrm{L}$. This parameter can be reasonably constrained in nature as our understanding of the spatial distribution of asperities and barriers improves through both short-term observations (e.g., coseismic slip and interseismic locking patterns) and longer-term geological indicators such as forearc basins, ridges, and peninsulas. Consequently, ${\mathrm{L}}_{\mathrm{b}}/\mathrm{L}$may serve as a useful parameter in future investigations aimed at identifying the factors that control the seismic behaviour of subduction megathrusts.

Although simplified, the combination of controlled laboratory experiments and quasi-dynamic numerical modelling in our study provides a powerful strategy to isolate the first-order effects of frictional heterogeneity on slip dynamics. While natural subduction zones are influenced by a complex interplay of additional factors—such as fluid pressure, sediment composition, and detailed plate geometry—our approach intentionally focuses on the fundamental role of along-strike segmentation. The convergence of these two distinct methodologies on a consistent, dimensionless parameter underscores the robustness of this geometric partitioning as a primary control on rupture propagation. This provides a foundational scaling relationship for fault segments characterized by barrier-asperity segmentation, establishing a benchmark that future studies using 3D models and incorporating additional physical complexities can now refine.

## Materials and methods

### Laboratory-based analogue experiments

#### Laboratory-based experimental set-up for stick-slip instability

The laboratory-based experiments were conducted at constant room temperature (~ 20 °C) conditions, considering a single-degree freedom spring-block model, which is the simplest model that reproduces the earthquake cycle, including megathrusts and slow earthquakes. The stick-slip instability, details laboratory-based experimental setup and its different components are presented in Fig. 1b (and Supplementary Table S1). We use a block on sandpaper setup, where a cuboid marble sample (5.0 × 5.0 × 1.5 cm^3^; surface roughness parameters are presented in the supporting document Table S1) placed over the frictional surface (sandpaper) is attached to a force sensor (S-type miniature load cell, capacity 1 kg) that is connected to the linear slider by a sample holder. The linear slider is attached to the computer-controlled servo motor system, which pushes the sample with a constant loading velocity (2 μm/s) over the frictional surface.

This experimental setup produces a characteristic sawtooth pattern in the force-time data, representing the earthquake cycle. During the stick phase, the motor-driven displacement causes elastic deformation of the force sensor, which has a defined stiffness due to its intrinsic design. This results in a linear increase in the measured force, directly quantifying the accumulation of shear stress on the frictional interface. The subsequent slip event is characterized by a rapid, near-instantaneous release of this stored elastic energy, observed as a sharp drop in the force reading. The shear force developing between the sample and the frictional surface interface during the stick phase and the instantaneous (or gradual) force drop during the seismic (or aseismic) slip phase is measured using the digital force sensor connected to a data acquisition system, which records the signal in terms of voltages. This recorded voltage is converted to force with ± 0.1 N precision. Before converting voltage to force, the load cell is calibrated by increasing the load and calculating the output signal at each step. From the load versus voltage graph, the load is estimated by multiplying the slope with the voltage value. The accelerometer on top of the sample records the instantaneous acceleration.

#### Framework for selecting surfaces with controlled frictional properties in laboratory experiments

Modern studies of friction^125–128^ have shown that “true” contact between two nominally flat surfaces consists of an ensemble of µm- scale contacts which collectively comprise only a small fraction of the apparent contact area. Chen et al.^129^ attributed the sticking phase to the locking of touching asperities (here, the term asperity indicates micro-scale contacts, different from the geological scale asperity in subduction zones) and the slipping phase to the brittle failure of these asperities and found that the fault asperities were as strong as the inherent strength of the host rock. In light of the preceding insights, we designed the contact interfaces for our experiments. Previous studies have used different setups to investigate seismic cycle characteristics, like gelatin on sandpapers^130^, polymethyl methacrylate (PMMA) sliding on sandpaper^131^, and PMMA-Teflon interfaces to create frictional contrast and stick-slip cycles^116^.

We model the frictional heterogeneities that may arise in nature from subducted seafloor features, without replicating their large-scale geometry. To establish a controlled frictional contrast, we used two different sandpapers (p2000 and p400) in our block-on-sandpaper setup. In this experimental framework, the small-scale surface roughness of the sandpapers serves as a measurable proxy for frictional behavior. Now, sandpapers have quasi-symmetric or isotropic roughness as indicated by the number of grits/inch^2^, such that the individual grit heights exhibit a truncated or skewed distribution. In other words, the heights and spacing of individual grits embedded in the sandpaper are more or less similar with less standard deviation. We quantified the surface roughness using standard parameters (Ra, Rq, Rz and Rt, detailed descriptions and schematic representation of these parameters are given in the supplementary information, Supplementary Figs. S1, S2, S3 and S4) measured with a surface roughness tester instrument for p2000, p400 and the sample surface (values of each parameter for the different surfaces is given in the Supplementary Table S1).

These parameters for p2000 (for example, Rq = 4 μm) and sample surface (Rq = 3.8 μm) are similar, resulting in a more or less homogeneous contact interface, greater micro contact interlocking and higher frictional resistance to slipping (Fig. 2, sample-p2000). The frictional behaviour of the p2000-sample interface was determined from a reference experiment where the entire fault area was p2000. As expected, the interface between these two surfaces behaved as an asperity contact, producing periodic or quasiperiodic stick-slip instability (discussed in detail in the previous section ). The dissimilarity of these parameters between the p400 (Rq = 10.7 μm) and the sample surface is significant. The morphology images (in Fig. 2) of the sample, p2000 and p400 surfaces were taken using JEOL JSM-6480 LV Scanning Electron Microscope (SEM) under analytical conditions of 2 μm beam diameter, 0.5 nA beam current, and 15 kV operating voltage. The SE images clearly reveal distinct differences in surface morphology, particularly in the degree and pattern of undulations across the different surfaces, which lead to differing frictional responses during the experiments with a frictionally heterogeneous interface. The stark contrast in surface textures between the sample surface and the p400 results in an interface that effectively behaves as a smooth surface in contact with a rough one. It is, therefore, intuitively apparent that such an interface would experience far less interlocking and weaker frictional strength, mimicking barrier characteristics (i.e. not an asperity) during a laboratory experiment.

This contrast in frictional strength serves as a proxy for the natural spectrum of interseismic coupling, which describes how both frictionally locked and creeping portions of a fault contribute to its overall deformation. In natural settings, the coupled area inferred from surface deformation is typically wider than the truly locked region, as creeping or velocity-strengthening patches remain mechanically linked to adjacent locked asperities and influence the overall coupling behaviour. In our laboratory configuration, the locking occurs along the interface between the solid block and the frictional surface. The p2000 region in contact with the block represents a strongly locked, velocity-weakening asperity, whereas the p400 region corresponds to a creeping or velocity-strengthening patch that exhibits reduced locking. The coexistence of these contrasting zones decreases the effective interseismic coupling of the system as a whole, consistent with the interplay of locked and creeping areas along natural megathrusts^132^. The frictional behaviour of the p400-sample interface was determined from an experiment (used as a reference) where the entire fault area was p400 (discussed in the results section). The relatively homogeneous contact between the sample and p2000 resulted in a greater real contact area (Fig. 2), unlike the sample and p400 interface. The mechanical response of the system—comprising the elastic force sensor, the rigid block and the frictional interface—together determines the slip behaviour. This response is a direct physical manifestation of the inherent frictional properties of the interface, where velocity-weakening regions promote seismic stick-slip and velocity-strengthening regions lead to aseismic slip. Figure 3 explains the characteristics of key laboratory experiments conducted for this study. Over an asperity (grey area, Rq = 4 μm) surface, we have introduced different types of barriers (green, Rq = 10.7) as frictional heterogeneities. We carried out 2 different sets of experiments to determine how the frictional heterogeneity influences the stick-slip cycle and characteristics: (1) single circular barriers over asperity, the size of which was increased systematically from experiments M_1_ to M_6_ and (2) Multiple circular barriers of different geometry and distribution.

### Quasi-DYNamic simulation

Earthquake cycle simulations in this study were performed using the boundary element code QDYN. It models tectonic fault slip under the quasi-dynamic approximation, which combines quasi-static elasticity with radiation damping^133–135^. QDYN employs adaptive time stepping to resolve both seismic and aseismic slip during earthquake cycles and can simulate slip on planar and non-planar faults in two- and three-dimensional elastic media as well as spring–block systems with implementation of rate and state friction laws.

#### Constitutive rate and state friction law

Laboratory-derived rate-and-state friction laws^112,136^ have been successfully used to incorporate experimental observations, accounting for the dependence of strength on slip velocity and evolving state variables that characterise asperity contacts. These laws explain both stable and unstable sliding between elastic solids^136–138^ and allow for strength loss during rapid slip, followed by rehealing, enabling repetitive failures. They have been successfully applied to model various earthquake phenomena, including nucleation, dynamic rupture propagation, postseismic slip, interseismic restrengthening, and aftershocks^65,133,134,139–142^. Additionally, they have been used to study the complexities of spatio-temporal slip behavior and interactions between ductile and brittle crustal regions^143–145^. We adopt the laboratory-derived rate-and-state friction laws, formulated with a single state variable to capture slip history dependence, assume constant normal stress $\sigma$ and follow the aging law proposed by Dieterich^112^ and Ruina^136^:

1 $$\left.\begin{array}{c}\tau=\left(\sigma-p\right)\left[{\mu}_{0}+aln\left(\frac{V}{{V}_{*}}\right)+bln\left(\frac{{V}_{*}\theta}{{D}_{c}}\right)\right]\\\frac{d\theta}{dt}=1-\frac{V\theta}{{D}_{c}}\end{array}\right\}$$

where $\tau$ is the shear stress, *V* is the slip velocity, *p* is the pore pressure, $\theta$ is the state variable, ${D}_{c}$ is the characteristic slip distance required for evolution to the steady state, ${\mu}_{0}$ is the value of the friction coefficient corresponding to the reference slip rate *V*_*0*_, and a > 0 and b are the constitutive parameters. The state variable *θ* is usually chosen in such a way that at steady state ${\theta}_{ss}=\frac{{D}_{c}}{V}$ and the shear stress $\tau$ evolves to its steady state value:

2 $${\tau}_{ss}=\left(\sigma-p\right)\left[{\mu}_{0}+\left(a-b\right)\mathrm{ln}\frac{V}{{V}_{0}}\right]$$

According to law (1), fault behaviour at steady state is defined by two conditions: VS for regions if $\left(a-b\right)>0$ i.e. $d{\tau}_{ss}/dV>0$ leading to stable slip and VW if $\left(a-b\right)<0$ i.e. $d{\tau}_{ss}/dV<0$, which are regions exhibiting seismogenic slip.

#### Quasi-DYNamic two-dimensional fault model

We consider a 2-D antiplane (Mode III) fault model, in which a 1-D fault is embedded in a 2-D uniform, elastic isotropic medium. The fault plane coincides with the x-z plane (Fig. S7) of a Cartesian coordinate system xyz. On the fault plane, purely dip-slip motion is assumed and only variations parallel to x-axis (along the strike) are considered. The only nonzero displacement is ${u}_{x}\left(y,z,t\right)$ and we define slip $\delta\left(z,t\right)$ on the fault plane as the displacement discontinuity $\delta\left(z,t\right)={u}_{x}\left({0}^{+},z,t\right)-{u}_{x}\left({0}^{-},z,t\right)$. The relevant shear stress on the fault plane is denoted by $\tau\left(z,t\right)={\sigma}_{xy}\left(0,z,t\right)$. It is possible to express the stress on the fault plane in terms of the slip history on the fault plane only^146,147^ as

3 $$\tau\left(z,t\right)={\tau}^{o}\left(z,t\right)+f\left(z,t\right)-\frac{\mu}{2c}V\left(z,t\right)$$

where, $\mu$ is the shear modulus, *c* is the shear wave speed, $V\left(z,t\right)=\dot{\delta}\left(z,t\right)=\partial\delta\left(z,t\right)/\partial\: t$ is the slip rate, ${\tau}^{o}\left(z,t\right)$ is the loading stress (i.e. stress that would act if the fault plane y = 0 were constrained against any slip) and $f\left(z,t\right)$ is a inear functional of prior slip over the causality cone and incorporates most of the electrodynamic response. The term $\frac{\mu}{2c}V\left(z,t\right)$ represents the radiation damping term (energy radiated by waves in the medium)^148^. Explicit extraction of that term from the functional f (z, t) avoids singularities of the convolution integrals^149^. The details of the elastodynamic solution and simulation methodology can be found elsewhere^133^.

## Supplementary Information

Below is the link to the electronic supplementary material.

Supplementary Material 1

## Data Availability

The datasets used and/or analysed during the current study are available from the corresponding author on reasonable request.
