# Supplementary material for: Effects of plate interface frictional heterogeneities on earthquake cycle dynamics in subduction zones
Source: Sci Rep. 2026 Apr 1;16:15396. doi: 10.1038/s41598-026-43399-7 (PMC13183892; doi:10.1038/s41598-026-43399-7)
Supplement: Supplementary file 1 — Supplementary Material 1 [file 41598_2026_43399_MOESM1_ESM.docx]

**Supplementary Information**

**of**

**Effects of plate interface roughness and frictional heterogeneities Earthquake Cycle Dynamics in Subduction Zones**

**Sayak Ray^1^, Abhijit Ghosh^2*^, Bhaskar Kundu^1*^, Batakrushna Senapati^3^**

^1^Department of Earth and Atmospheric Sciences, NIT Rourkela, Rourkela, India, 769008.

^2^Department of Earth Sciences, University of California-Riverside, 900 University Ave, Riverside, CA 92521, USA.

^3^Department of Earth Science, National Central University, No. 300, Jhongda Rd., Chungli, Taoyuan 320, Taiwan.

***Corresponding authors: Abhijit Ghosh,** Department of Earth Sciences, University of California-Riverside, 900 University Ave, Riverside, CA 92521, USA ([aghosh.earth@gmail.com](mailto:aghosh.earth@gmail.com)); **Bhaskar Kundu**, Department of Earth and Atmospheric Sciences, NIT Rourkela, Rourkela-769008, India, (email: [rilbhaskar@gmail.com](mailto:rilbhaskar@gmail.com)).

**The contents include:**

1. Table S1 - Parameters for laboratory experiments.
2. Details of Surface Roughness parameters
3. Schematic representation of L_b_/L
4. Results of continuous linear barrier experiments
5. Schematic representation of xz-plane of a fault with alternating VS and VW patches
6. Table S2: Parameters for QDYN Numerical Simulation for Fig. 8, 9 and 10.
7. **Table S1:** Parameters for laboratory experiments.

| Parameters | | Values | | |
| --- | --- | --- | --- | --- |
| Normal Load of the sample | | 0.5N | | |
| Area of the sample (contact area) | | 25 cm^2^ | | |
| Force sensor (capacity) | | 1 kg | | |
| Accelerometer (frequency) | | 201 Hz | | |
| Loading Velocity (V_L_) | | 2 µm/s | | |
| Roughness parameters (µm) | R_a_ | 3.68 ± 0.39 | 3.97 ± 0.03 | 10.56 ± 0.99 |
|  | R_q_ | 3.82 ± 0.45 | 4.02 ± 0.11 | 10.68 ± 0.94 |
|  | R_z_ | 10.18 ± 0.60 | 10.96 ± 0.22 | 29.87 ± 2.79 |
|  | R_t_ | 10.28 ± 0.75 | 11.27 ± 0.15 | 30.16 ± 2.81 |
| *Frictional Surfaces* | | *Sample* | *p2000(asperity)* | *p400(barrier)* |

1. **Roughness parameters:**

Surface roughness is a critical parameter in characterizing the topography of a material's surface, influencing its mechanical properties, frictional behaviour, and contact interactions. It provides quantitative insights into surface texture, which can affect adhesion, wear resistance, and tribological performance. Maximum Profile Peak Height, Rp, the distance between the highest point of the profile and the mean line (M) within the evaluation length (L). Maximum profile valley depth, Rv, the distance between the deepest valley of the profile and the mean line (M) within the evaluation length (L):

**
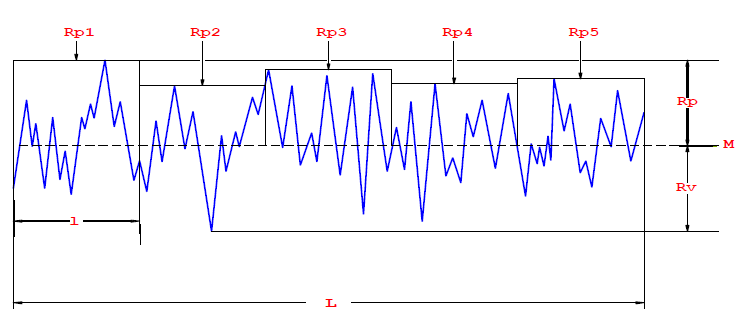
**

**Fig. S1:** Schematic representation of Maximum Profile Peak Height (Rp) and Maximum profile valley depth (Rv) with respect to the Mean line (M, dotted line) in an evaluation length (L).

Various roughness parameters are used to describe different aspects of surface irregularities, each capturing specific features of the surface profile. The following parameters are commonly used to assess surface roughness:

1. **Roughness average** (**R_a_**) is the arithmetic average of the absolute values of the profile heights (h) over the evaluation length.

$$R_{a}= \frac{1}{n}\sum_{i=1}^{n} {Rp}_{i}$$

2. **R_q_** is the **Root mean square average** of the profile heights (h) over the evaluation length

$$R_{q}= \sqrt{\frac{{Rp}_{1}^{2}+{Rp}_{2}^{2}+{Rp}_{3}^{2}\ldots\ldots\ldots\ldots+{Rp}_{n}^{2}}{n}}$$

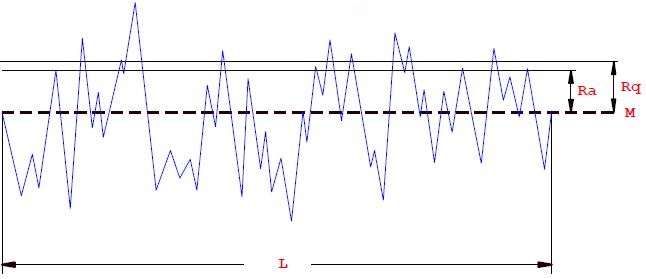


**Fig S2:** Schematic representation of R_a_ and R_q_.

1. **Ten Point Height of Irregularities (R_z_):** the average value of the absolute values of the heights of five highest profile peaks and the depths of five deepest valleys within the evaluation length.

$$R_{z}= \frac{1}{5}\sum_{j=1}^{5} \left( R_{P_{j}}+R_{V_{j}} \right)$$

$$R_{z}=average maximum height of the profile$$

$$R_{P_{j}}=height of peaks$$

$$R_{V_{j}}=depth of valleys$$

$R_{v_{j}}$ +


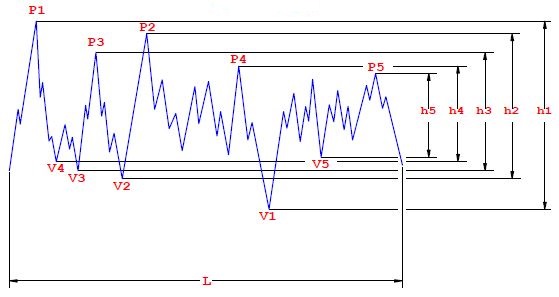


**Fig S3:** Schematic representation of R_Z_

1. **Maximum Heights within a Sampling Length (R_t_):** the vertical distance between the highest and lowest points of the profile within a sampling length.


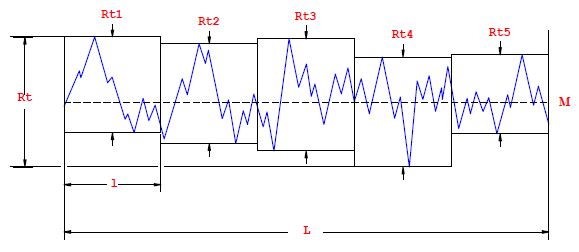


**Fig. S4:** Schematic representation of R_t_.

1. Schematic representation of L_b_/L

**
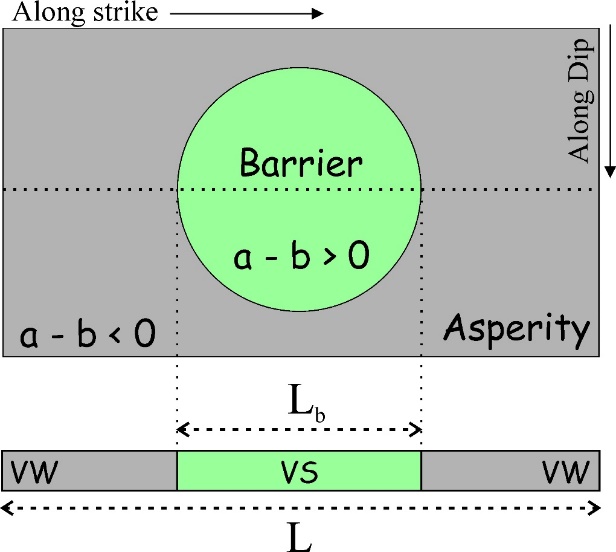
Figure S5:** Schematic representation of the diameter of the barrier (L_b_) and along-strike length of the sample contact surface (L).

1. Results of continuous linear barrier experiments


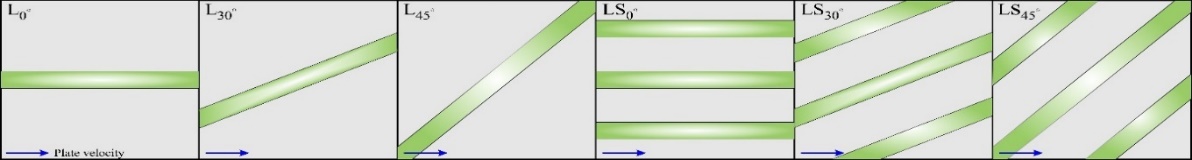

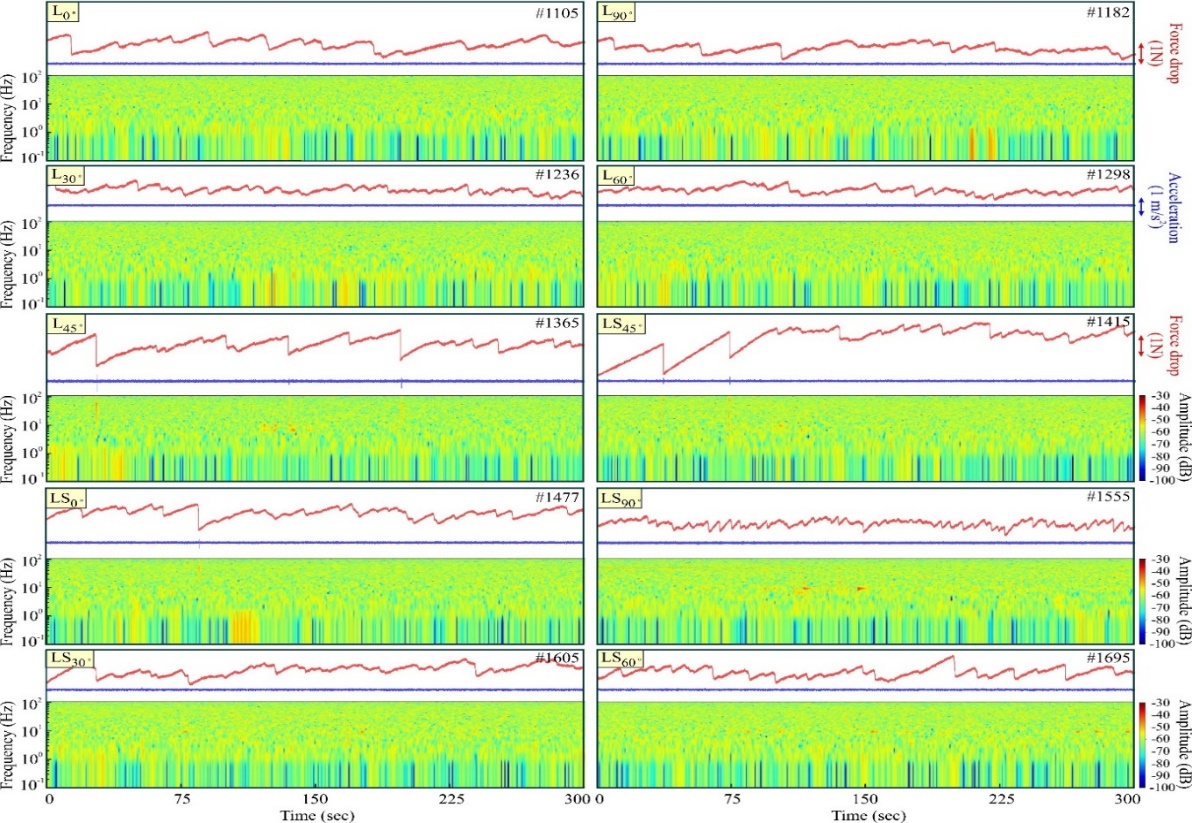


**Figure S6:** Results of single (L_θ_) and multiple (LS_θ_) linear barrier stick-slip experiments. Shear stress (in red) and acceleration (in blue) are recorded from the force sensor and accelerometer data, respectively, and their corresponding spectrogram of each experiment are plotted for 200s of experimental run time. The experiment number is mentioned in the top right corner of each plot.

1. Schematic representation of xz-plane of a fault with alternating VS and VW patches


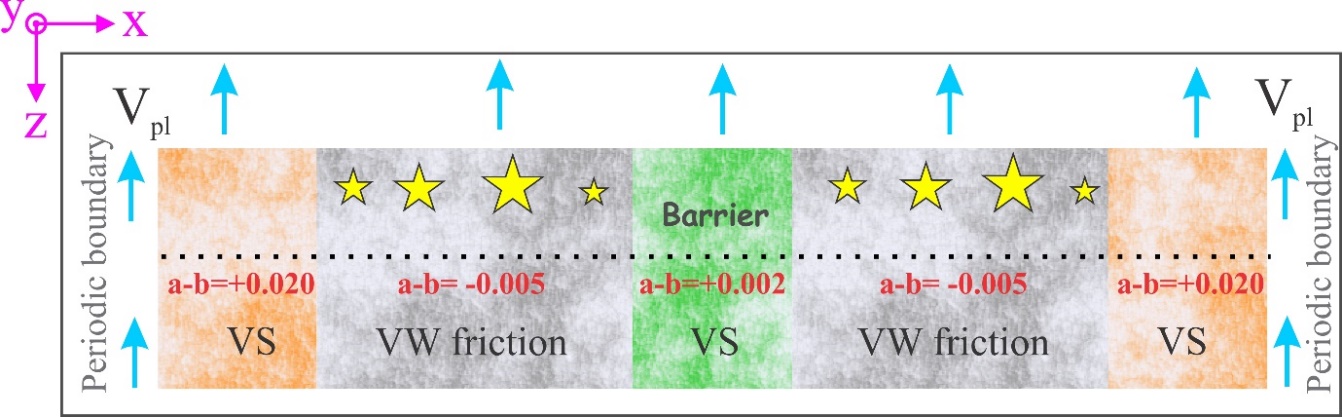


**Figure S7:** Schematic representation VS barrier (green) and VW (grey) patches on either side, bounded by VS buffer zones (orange). Earthquakes (yellow stars) occur in the VW region. Values of the rate and state parameters are shown in red.

1. **Table S2:** Parameters for QDYN Numerical Simulation for Fig.8, 9 and 10.

| Region | Parameters | Symbols | Values |
| --- | --- | --- | --- |
| Synthetic Fault simulation | Fault length along the strike (including buffer zone, in km) | L | 305/310/325/ 350/375/400/450/500 |
|  | Shear modulus (GPa) | µ | 32 |
|  | Shear wave speed (km/s) | C_s_ | 3.4 |
|  | Plate velocity (mm/year) | V_pl_ | 50 |
|  | Characteristic slip length (m) | D_c_ | 0.004 |
|  | Effective normal stress (MPa) | $\bar{\sigma}$ | 25 |
|  | Size of the VS segment (km) | D_VS_ | 5/10/25/50/75/100/150/200 |
|  | Size of the VW segment (left/right of the VS segment) (km) | D_VW1_ D_VW2_ | 100 100 |
|  | Size of the two VS buffer zones (km) | B_VS_ | 100 |
|  | Rate and state parameter a-b in VW | (a-b)_VW_ | -0.005 |
|  | Rate and state parameter a-b in VS | (a-b)_VS_ | +0.002 |
|  | Rate and state parameter a-b in the VS buffer zones on both the side | (a-b)_B_ | +0.020 |
|  | Reference slip velocity (m/s) | V_0_ | 10^-6^ |
|  | Reference friction coefficient | f_0_ | 0.6 |
|  | Cell size (m) | ∆x | 141 |
| Alaska Aleutian Subduction zone simulation | Fault length along strike (including buffer zone, in km) | L | 910 |
|  | Shear modulus (GPa) | µ | 32 |
|  | Shear wave speed (km/s) | C_s_ | 3.4 |
|  | Plate velocity (mm/year) | V_pl_ | 57 |
|  | Characteristic slip length (m) | D_c_ | 0.004 |
|  | Effective normal stress (MPa) | $\bar{\sigma}$ | 50 |
|  | Size of the VS segment (km) | D_VS_ | 270 |
|  | Size of the VW segment (left/right of the VS segment) (km) | D_VW1_ D_VW2_ | 160 280 |
|  | Size of the two VS buffer zones (km) | B_VS_ | 100 |
|  | Rate and state parameter a-b in VW | (a-b)_VW_ | 40 |
|  | Rate and state parameter a-b in VS | (a-b)_VS_ | 71.5 |
|  | Rate and state parameter a-b in the VS buffer zones on both the side | (a-b)_B_ | 67.5 |
|  | Reference slip velocity (m/s) | V_0_ | 76.5 |
|  | Reference friction coefficient | f_0_ | 0.6 |
|  | Cell size (m) | ∆x | 141 |
| Himalaya Deformation Front simulation | Fault length along strike (including buffer zone, in km) | L | 535 |
|  | Shear modulus (GPa) | µ | 32 |
|  | Shear wave speed (km/s) | C_s_ | 3.4 |
|  | Plate velocity (mm/year) | V_pl_ | 40 |
|  | Characteristic slip length (m) | D_c_ | 0.004 |
|  | Effective normal stress (MPa) | $\bar{\sigma}$ | 25 |
|  | Size of the VS segments (left to right, in km) | D_VS_ | 15/20/20 |
|  | Size of the VW segment (left to right, in km) | D_VW_ | 125/120/85/100 |
|  | Size of the two VS buffer zones (km) | B_VS_ | 25 |
|  | Rate and state parameter a-b in VW | (a-b)_VW_ | -0.005 |
|  | Rate and state parameter a-b in VS | (a-b)_VS_ | +0.002 |
|  | Rate and state parameter a-b in the VS buffer zones on both the side | (a-b)_B_ | +0.020 |
|  | Reference slip velocity (m/s) | V_0_ | 10^-6^ |
|  | Reference friction coefficient | f_0_ | 0.6 |
|  | Cell size (m) | ∆x | 141 |
